# Trans‐omic profiling between clinical phenoms and lipidomes among patients with different subtypes of lung cancer

**DOI:** 10.1002/ctm2.151

**Published:** 2020-08-20

**Authors:** Zhenhua Zhu, Linlin Zhang, Jiapei Lv, Xiaoxia Liu, Xiangdong Wang

**Affiliations:** ^1^ Institute of Clinical Science, Zhongshan Hospital, Shanghai Medical College Fudan University Shanghai China; ^2^ Shanghai Institute of Respiratory Diseases, Zhongshan Hospital, Shanghai Medical College Fudan University Shanghai China

**Keywords:** lipidomics, lung cancer, phenomes, subtypes, trans‐omics

## Abstract

Lung cancer has high mortality, often accompanied with systemic metabolic disorders. The present study aimed at defining values of trans‐nodules cross‐clinical phenomic and lipidomic network layers in patients with adenocarcinoma (ADC), squamous cell carcinomas, or small cell lung cancer (SCLC). We measured plasma lipidomic profiles of lung cancer patients and found that altered lipid panels and concentrations varied among lung cancer subtypes, genders, ages, stages, metastatic status, nutritional status, and clinical phenome severity. It was shown that phosphatidylethanolamine elements (36:2, 18:0/18:2, and 18:1/18:1) were SCLC specific, whereas lysophosphatidylcholine (20:1 and 22:0 sn‐position‐1) and phosphatidylcholine (19:0/19:0 and 19:0/21:2) were ADC specific. There were statistically more lipids declined in male, <60 ages, late stage, metastasis, or body mass index < 22 . Clinical trans‐omics analyses demonstrated that one phenome in lung cancer subtypes might be generated from multiple metabolic pathways and metabolites, whereas a metabolic pathway and metabolite could contribute to different phenomes among subtypes, although those needed to be furthermore confirmed by bigger studies including larger population of patients in multicenters. Thus, our data suggested that trans‐omic profiles between clinical phenomes and lipidomes might have the value to uncover the heterogeneity of lipid metabolism among lung cancer subtypes and to screen out phenome‐based lipid panels as subtype‐specific biomarkers.

## Graphical abstract

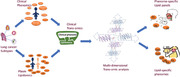

## 1 INTRODUCTION

Lung cancer is a systemic and aggressive disease with high morbidity and mortality, and it is often accompanied with systemic metabolic disorders, for example, up‐ or downregulated expression of mechanism‐associated genes or activation of metabolism‐dependent enzymes. For example, metabolism‐associated genes of small cell lung cancer (SCLC) cells altered after mitogen‐activated protein kinase (MAPK) kinase module MEK5/ERK5 was blocked, accompanied by dysfunctions of several lipid metabolism pathways like the mevalonate pathway for cholesterol synthesis.^1^ Lipids, mainly including subclasses of phosphatidic acid (PA), phosphatidylcholines (PC), phosphatidylethanolamine (PE), phosphatidylglycerol (PG), phosphatidylinositol (PI), and phosphatidylserine (PS), have multiple important biological functions, such as biomembrane composition, vesicular trafficking, adhesion, migration, apoptosis, energy storage, neurotransmission, signal transduction, and posttranslational modification. They have alterations under circumstance of lung cancer. Circulating levels of PCs and PEs in patients with nonsmall cell lung cancer (NSCLC) differed from those with noncancer lung diseases or health and were suggested as diagnostic biomarkers of early NSCLC.^2^ The heterogeneity of circulating lipidomic profiles was found to exist among patients with squamous cell carcinomas (SCC), adenocarcinoma (ADC), or SCLC, and there was a clear correlation between genomic and lipidomic profiles of lipid‐associated proteins and enzymes.^3^ As the part of clinical trans‐omics, the lung cancer‐specific and subtype‐specific lipidomics in the circulation were defined and evidenced by integrating lipidomic data with genomic expression of lipid proteins among lung cancer subtypes.

Clinical trans‐omics is defined as a new subject to integrate clinical phenomes with molecular multi‐omics for understanding molecular mechanisms of diseases in multiple dimensions.^4^ Clinical trans‐omics becomes more important as a new and novel approach for the discovery of disease‐specific biomarkers and therapeutic targets, although there are still many obstacles to be overcome, for example, specificity and decisive role of trans‐nodules among multi‐omic networks for intra‐ and intercellular communication.^5^, ^6^ Recent studies applied the trans‐omics among phosphor‐proteomics, transcriptomics, gene sequencing, and genomics for new molecular category of liver cancer, to provide a new therapeutic strategy.^7^ As the part of clinical trans‐omics, clinical lipidomics was considered as one of major metabolic profiles for identification and validation of lung cancer‐specific biomarkers by integrating clinical phenomes with lipidomic profiles.^8^, ^9^ Clinical lipidomics could demonstrate the complexity of the lipidome in metabolic diseases and lung cancer and presented the variation among diseases and subtypes of lung cancer.^10^, ^11^, ^12^

Our previous study demonstrated the difference of lipidomic profiles among patients with different lung cancer subtypes and the potential association between lipidomic phenotypes and gene expression of lipid metabolism‐associated proteins and enzymes as a concept evaluation.^3^ The present study furthermore investigated the values of trans‐nodules cross‐clinical phenomic and lipidomic network layers in the recognition of lung cancer subtypes (ADC, SCC, and SCLC), in order to understand clinical phenome‐associated lipid changes or lipidomic phenotype‐associated clinical phenomes. We also evaluated the differences of lipidomic profiles between male and female, various ages, early and late stages, with or without metastasis, body mass index (BMI) <22 or >22, and digital evaluation scores less or more than 90.

## 2 METHODS AND MATERIALS

### 2.1 Chemical agents

The internal standard cocktails were subscribed from Avanti Lipids Polar (Alabaster, AL, USA); the acetone, acetonitrile, ammonium bicarbonate, dithiothreitol, formic acid, iodoacetamide, and Tris base (Analytical Grade) from Sigma‐Aldrich (St. Louis, MO, USA); and ammonium acetate (NH4OAc), hexane, isopropyl alcohol (IPA), methanol, and high‐performance liquid chromatography‐grade chloroform (CHCl3) from Merck Millipore (Billerica, MA, USA).

### 2.2 Patient population

The study, designed as a case‐control approach, was approved by the Ethical Evaluation Committee of Zhongshan Hospital (ethical code B2018‐187). The subjects gave informed consent for clinical data collection and lipids analysis before all the other procedures. The study included 54 lung cancer patients diagnosed according to pathology, of whom 28 were ADC, 15 SCC, 11 SCLC, and 15 other healthy people. The stage and severity of lung cancer were defined according to the Eighth Edition of TNM Classification for Lung Cancer.^13^ Patients were recruited during October 2016 to March 2017. Healthy controls participated were blood donors in Zhongshan Hospital. Subjects with other respiratory diseases or family history of lung cancer were excluded. Fasting blood was drawn from healthy controls and lung cancer patients on the day of entering hospital to harvest plasma. All the clinical data, including symptoms, signs, laboratory tests, images, pathologic information, and survival status 3 years later, were collected and followed up.

### 2.3 Digital evaluation score system

The Digital Evaluation Score System (DESS) is a score index system by which clinical descriptive information of each phenome can be translated into clinical informatics.^14^ When the severity of each component was scored as 0, 1, 2, or 4, of which 4 represented the most severe condition, whereas 0 indicated normal physiological range. The gross DESS scores ranged from 0 to 584 points; the higher the score, the severer the condition. A total of 194 clinical phenomes were collected and scored in each of three lung cancer group, including 30 histories, 30 symptoms, 18 signs, 27 laboratory measurements, 49 image features, and 40 pathologic indexes, as listed in Table S1.

### 2.4 Lipid extraction for mass spectrometry analysis

About 200 μL plasma was collected into a glass tube, into which 10 μL internal standard was added and then 5 mL of methanol:chloroform:formic acid (10:10:1), as reported previously.^3^, ^15^ This mixture was incubated at –20°C overnight after vigorous shaking. Two milliliters of Hajra's reagent (0.2 M H_3_PO_4_, 1 M KCl) were dropped, blended, and centrifuged at 3000 rpm for 5 min. After stratification, chloroform in the lower layer was pipetted to another glass tube and concentrated to 200 μL with the nitrogen flow, where the liquid with isopropyl alcohol:hexane:100 mM ammonium acetate at the ratio of 58:40:2 was added till 1 mL. The sample was then centrifugated at 14 000 rpm at 4°C for 20 min. The normal‐phase liquid chromatography coupled Triple‐Quadrupole mass spectrometer (QTRAP 6500, SCIEX, Framingham, MA, USA) was used for lipid extraction by the positive and negative electrospray ionization mode. In the multiple reaction, Q‐Trap was utilized to scan the precursor/product ion and examine the mode operation. Each test was repeated three times. The peak area of each pair was quantified with multiple reaction monitoring data by the software MultiQuant (AB SCIEX).

### 2.5 Purification of plasma lipids

Lipid samples were derived through Ultimate SiO_2_ (250 mm × 4.6 mm, 5 μm; Agilent Technologies, Santa Clara, CA, USA) with 1.5 mL/min flow rate, high‐purity helium. In the meanwhile, 2.0 μL was added with the split ratio of 50:1 at the ignition chamber temperature of 220°C and the injection port temperature of 150°C. It was started at temperature 150°C, which gradually increased (4°C/min) to 250°C, and kept for 5 min. The mass spectrometry was subjected to liquid chromatography‐mass spectrometer analysis (FOCUS DSQTM II, Thermo Fisher Scientific), mainly under the following conditions: Electron Ionization (EI) as ionization source, ion source temperature at 200°C, ionization voltage at 70 eV, multiplier voltage at 0.9 kV, 4 min solvent delaying, and 50‐650 amu of scan range.

### 2.6 Identification of lipidomic profiles

Lipid extracts were loaded onto an Ultremex silica column (250 mm × 4.6 mm, 5 μm), which was fitted with a 2 mm × 4 mm silica guard cartridge (Phenomenex, Torrance, CA, USA), and then eluted. The sample was enriched at a gradient of 300 nL/min. In the 50 min's run, B phase was 50% from 0 to 5 min, then rose to 100% from 5 to 30 min, linearly ramped for 10 min, as to return 50% from 40 to 41 min until the end. The Q‐Trap was conducted in the multiple‐reaction monitoring mode, and the different precursor/product ion pairs were scanned in the positive/negative electrospray ionization mode. Up to 502 lipids of plasma samples were carried out to get possible lipids chemical structures.

### 2.7 Comprehensive analyses of lipidomic profiles

MultiQuant software (AB SCIEX) was used to process data, after lipids were identified by mass spectrometry. Further, MetaboAnalyst software 4.0 (www.metaboanalyst.ca) was utilized for conducting multivariate statistical analysis, cluster analysis, dimensionality reduction, and making heat map.

### 2.8 Trans‐nodule analyses cross phenome and lipidome network layers

The type‐specific lipids were identified as more than two times elevated or declined significantly compared with other lung cancer subtypes (fold change > 2 and *P*‐value < .05), whereas the co‐expression lipids were identified as those similarly changed in all lung cancer subtypes as compared with healthy controls. The expression quantitative trait locus (eQTL) model was utilized to evaluate trans‐nodules between lipidomic profiles and clinical phenomes.

### 2.9 Statistical analysis

Data were presented as mean ± SE. The means of each group were used for calculation and comparison. Statistical significance of differences between two groups or among multiple groups was determined by Student's *t*‐test or one‐way ANOVA test, respectively. Statistical significance was affirmed when *P*‐value < .05. We also separately calculated mean values of each phenome's DESS score in different lung cancer subtypes, which were ranked to obtain top 10 clinical phenomes of those three groups of patients. Volcano maps showed the significantly elevated or declined lipids in ADC, SCC, or SCLC patients. A VIP plot was further exploited to sort the lipids according to their importance to differentiate the four groups. To explore the correlation between lipid elements and clinical phenomes, we applied the lipid‐quantitative trait loci model modified from eQTL model. Besides, MatrixlQTL R package was used to acquire the significant phenome‐lipid pairs and corresponding *P*‐values. Moreover, GraphPad Prism was utilized to make the receiver operating characteristic curve to evaluate the early‐diagnostic value and accuracy of clinical phenome‐specific lipid elements in ADC, SCC, or SCLC. The present study furthermore analyzed the significant differences of lipids among different ages (eg, <60, 60‐70, and >70), between female and male, early and late stage, metastasis and non‐metastasis, high and low DESS scores (≤90 and >90), and high and low BMI (≤22 and >22).

## 3 RESULTS

### 3.1 Clinical phenomes of patients with lung cancer subtypes

Eighteen female and 36 male lung cancer patients were enrolled in the present study, aged from 43 to 80 (63 ± 10) years old, including 28 ADC, 15 SCC, and 11 SCLC. The total scores of DESS were 72 ± 28, 79 ± 25, and 98 ± 19 in patients with ADC, SCC, and SCLC, respectively. The DESS values of SCLC group were significantly higher than those of healthy control group (*P* < .05). Top 10 clinical phenomes of ADC, SCC, or SCLC patients as well as patients survived or nonsurvived during study period were listed in Table 1. Stages at primary diagnosis and recruitment period for the study, lymphatic metastasis (N1‐2) in ipsilateral paratracheal hilum or mediastinum, and enhanced images (eg, focus enhanced in CT or hypermetabolism in PET/CT) were shown in all three subtypes of lung cancers. In addition, thyroid transcription factor‐1 (TTF‐1), Napsin A, keratin 7, and location of tumor were noticed in ADC; obscure boundary, emphysema, tumor size, the cycle number of first line chemotherapy, obstructive pneumonia atelectasis, and pulmonary nodule in SCC; as well as number of metastatic lymph nodes in SCLC, separately. Top 10 clinical phenomes were similar between survived and nonsurvived patients, but the total amounts of DESS of nonsurvived patients were significantly higher than those of survived patients (Table 1). Of 194 total clinical phenomes, 46 had the statistical significance of each two groups in‐between (Table 2; *P* < .05 or less).

**TABLE 1:** Top 10 clinical phenomes of patients with adenocarcinoma (ADC), squamous cell carcinomas (SCC), or small cell lung carcinoma (SCLC) as well as lung cancer patients survived or nonsurvived

Patients with ADC	Patients with SCC	Patients with SCLC	Patients survived	Patients nonsurvived
Stage at primary diagnosis	N1 (ipsilateral hilum)	Stage at recruitment time	Stage at primary diagnosis	Stage at recruitment time
3.57 ± 0.19	3.2 ± 0.43	3.82 ± 0.18	3.00 ± 0.45	3.90 ± 0.10
Stage at recruitment time	Enhanced image	Stage at primary diagnosis	N2 (ipsilateral mediastinum)	Stage at primary diagnosis
3.46 ± 0.21	3.2 ± 0.43	3.82 ± 0.18	2.80 ± 0.61	3.90 ± 0.10
TTF‐1	Stage at recruitment time	N1 (ipsilateral Paratracheal)	Enhanced image	N1 (ipsilateral paratracheal)
3.43 ± 0.27	3.13 ± 0.39	3.64 ± 0.36	2.80 ± 0.61	3.43 ± 0.31
N2 (ipsilateral mediastinum)	Stage at primary diagnosis	T (tumor)	Stage at recruitment time	N2 (ipsilateral mediastinum)
3.14 ± 0.32	3.13 ± 0.39	3.27 ± 0.38	2.70 ± 0.47	3.43 ± 0.31
Napsin A	obscure boundary	N1 (ipsilateral hilum)	N1 (ipsilateral hilum)	N1 (ipsilateral hilum)
3.07 ± 0.28	2.13 ± 0.53	3.27 ± 0.49	2.40 ± 0.65	3.24 ± 0.35
Enhanced image	Emphysema	N2 (ipsilateral mediastinum)	TTF‐1	Enhanced image
2.71 ± 0.36	2.13 ± 0.53	3.27 ± 0.49	2.40 ± 0.65	3.05 ± 0.38
Location	T (tumor)	Enhanced image	Napsin A	N2 (below carina)
2.29 ± 0.38	1.87 ± 0.43	3.27 ± 0.49	2.20 ± 0.61	2.86 ± 0.40
N1 (ipsilateral paratracheal)	L1 cycle	pulmonary nodule	Location	TTF‐1
2.29 ± 0.38	1.87 ± 0.48	3.27 ± 0.49	2.00 ± 0.67	2.86 ± 0.40
N1 (ipsilateral hilum)	Obstructive pneumonia atelectasis	N (LN)	N1 (ipsilateral paratracheal)	N (LN)
2.29 ± 0.38	1.87 ± 0.53	3.00 ± 0.45	2.00 ± 0.67	2.81 ± 0.31
CK7	pulmonary nodule	Maintenance treatment	Lobular	T (tumor)
2.21 ± 0.37	1.87 ± 0.53	2.91 ± 0.56	2.00 ± 0.67	2.71 ± 0.36

Abbreviations: N, degrees of lung cancer metastasis to lymph nodes of TNM category; N1, degree 1 that has metastatic lymph nodes near pulmonary center and side of main bronchia; N2, degree 2 that has metastatic lymph nodes in the same side of the mediastinum as lung cancer; TTF‐1, thyroid transcription factor‐1 as an immunohistochemical biomarker for adenocarcinoma; CK7, keratin 7 as an immunohistochemical biomarker for epithelial cells; L1 cycle, number of the first line chemotherapy cycles.

**TABLE 2:** Comparisons of clinical phenomes in increased folds and statistical significance (*P*‐values) between each two groups of adenocarcinoma (ADC), squamous cell carcinoma (SCC), or small cell lung cancer (SCLC) patients

	ADC vs SCC		SCC vs SCLC		ADC vs SCLC	
	Folds	*P*‐values	Folds	*P*‐values	Folds	*P*‐values
TTF‐1	0.00	.00^*^	NA	.00^*^	0.74	.13
Napsin A	0.00	.00^*^	NA	NA	0.00	.00^*^
Bullae	NA	.00^*^	0.94	.90	NA	.00^*^
P40	NA	.00^*^	0.00	.01^*^	NA	NA
Hemoptysis	14.93	.00^*^	0.34	.07	5.09	.13
Emphysema	11.95	.00^*^	0.00	.00^*^	0.00	.45
Sputum	4.07	.00^*^	0.28	.00^*^	1.16	.85
CK7	0.00	.00^*^	NA	.01^*^	0.62	.22
Hb	5.60	.00^*^	0.76	.54	4.24	.04^*^
Cough	2.31	.00^*^	1.70	.13	0.73	.16
EGFR	0.00	.00^*^	NA	NA	0.00	.01^*^
Vacuole cavity	NA	.00^*^	0.00	.07	NA	NA
CEA	0.00	.01^*^	NA	NA	0.07	.03^*^
N2 (ipsilateral mediastinum)	0.51	.01^*^	2.05	.03^*^	1.04	.83
New metastasis	0.00	.01^*^	NA	.25	0.28	.15
P63	4.43	.02^*^	0.65	.45	2.86	.09
Cyfra211	3.53	.02^*^	0.24	.11	0.85	.81
Obstructive pneumonia atelectasis	3.27	.02^*^	0.97	.95	3.18	.04^*^
Smoking	2.21	.02^*^	0.79	.55	1.74	.25
Pleural pull	0.17	.02^*^	0.00	.40	0.00	.01^*^
Third‐line	5.60	.03^*^	0.00	.05	0.00	.45
WBC	5.60	.04^*^	2.27	.22	12.73	.00^*^
L1 cycle	2.01	.04^*^	1.27	.47	2.55	.00^*^
PD‐L1 tumor	4.85	.04^*^	0.00	.05	0.00	.45
PT	NA	.05^*^	0.00	.22	NA	NA
Bronchiectasis	NA	.05^*^	2.73	.49	NA	.11
PD‐1 tumor	NA	.05^*^	0.00	.22	NA	NA
L2 chemo regimen	0.00	.20	NA	.00^*^	27.15	.00^*^
Syn	NA	NA	NA	.00^*^	NA	.00^*^
Maintenance treatment	1.87	.39	3.64	.01^*^	6.79	.00^*^
NSE	0.93	.95	15.00	.01^*^	14.00	.00^*^
N1 (ipsilateral Paratracheal)	0.70	.29	2.27	.01^*^	1.59	.05^*^
CD56	1.17	.88	5.45	.02^*^	6.36	.00^*^
CHG	NA	NA	NA	.02^*^	NA	.00^*^
T (tumor)	1.34	.38	1.75	.03^*^	2.35	.00^*^
PD‐1 interstitial	3.11	.07	0.00	.03^*^	0.00	.27
Sum of all tumors (mm)	0.89	.76	1.98	.04^*^	1.77	.07
N (LN)	0.84	.46	1.73	.05^*^	1.45	.07
Ki‐67	1.99	.05	1.62	.24	3.22	.00^*^
Bronchial stenosis	2.49	.19	2.05	.16	5.09	.00^*^
N3 (opposite side)	1.49	.51	2.39	.06	3.56	.00^*^
Burr	0.31	.05	0.00	.22	0.00	.01^*^
Neu	3.73	.20	2.05	.39	7.64	.01^*^
L2 cycle	1.40	.56	2.12	.06	2.97	.02^*^
Pulmonary nodule	1.14	.73	1.75	.07	1.99	.02^*^
CK5/6	2.80	.22	4.09	.16	11.45	.02^*^

### 3.2 Lipidomic profiles of patients with lung cancer subtypes

**FIGURE 1:**
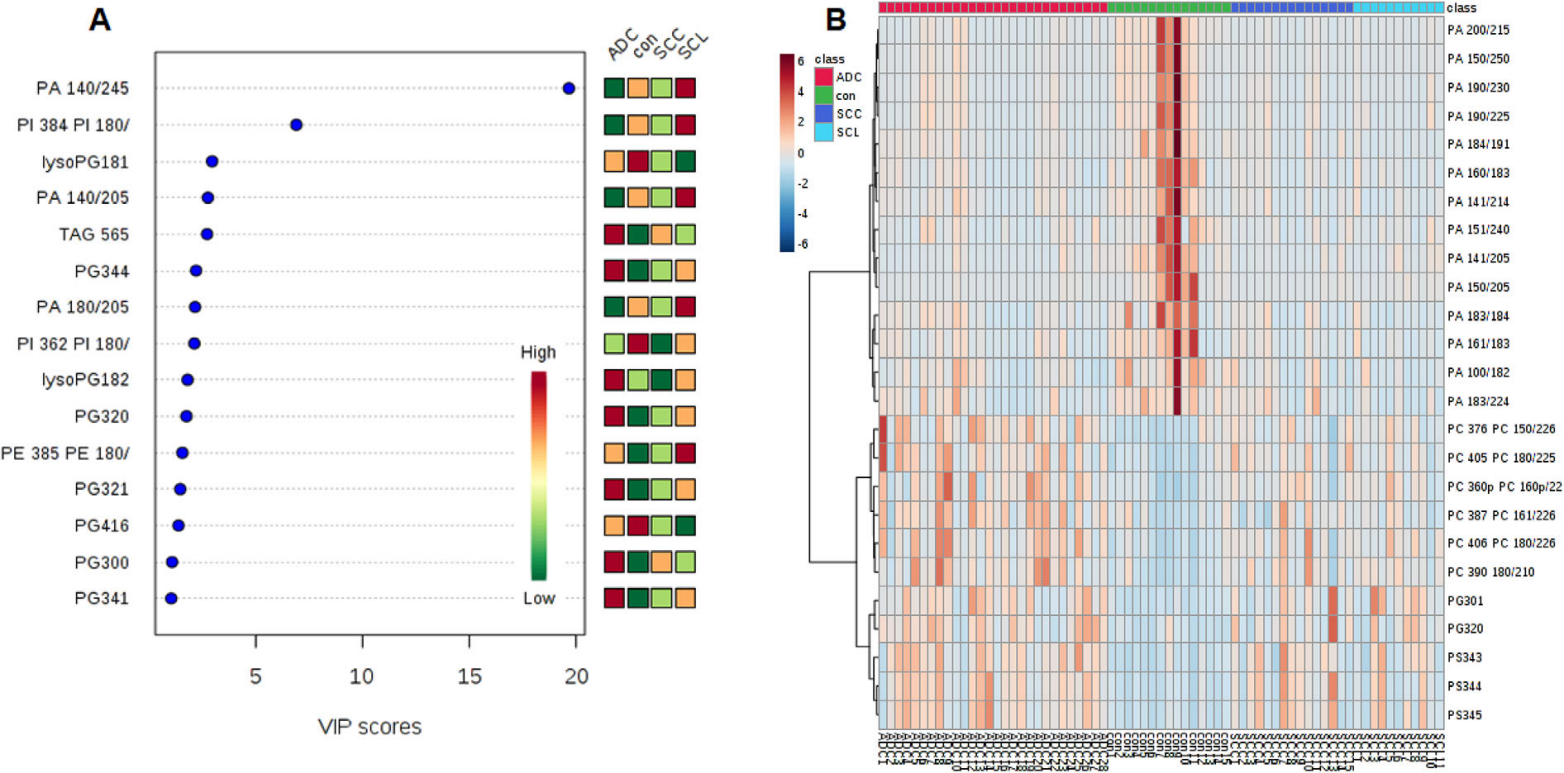
Scores of altered lipid elements in variable import in project (VIP) chart (A), where top 15 lipid elements were defined among patients with adenocarcinoma (ADC), squamous cell carcinomas (SCC), small cell lung cancer (SCL), and healthy controls (CON). The *x*‐axis represents the VIP score and the *y*‐axis represents the lipid elements corresponding to the VIP score. The right color grid stands for the relative concentration of lipid elements in four groups. The degree of altered concentrations increased from green to red. The heatmap (B) describes the top 25 lipid elements at the high concentration and the degree of lipid elements increased from blue (low) to brown (high)

**FIGURE 2:**
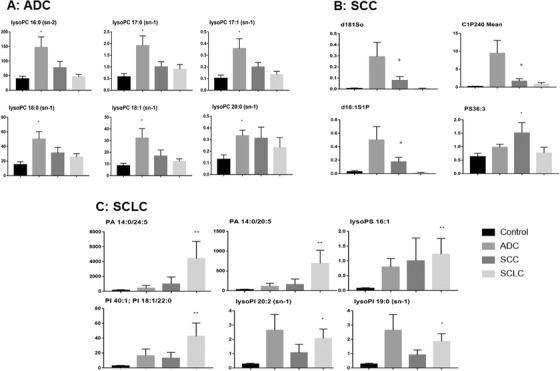
Top six significantly increased lipid elements in patients with ADC (A), SCC (B), and SCLC (C). * and ** stand for the *P*‐value less than .05 and .01, respectively, as compared with the healthy control

**FIGURE 3:**
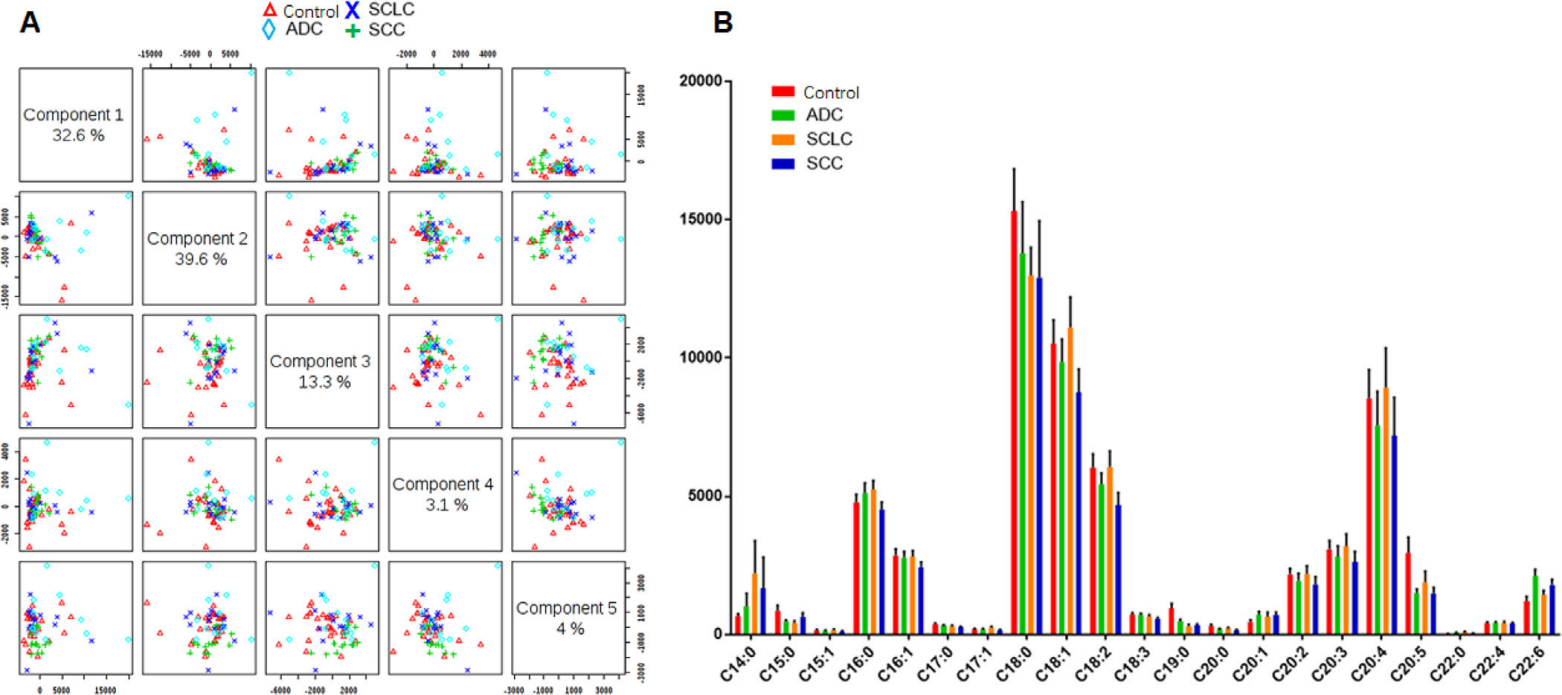
Histography of five component distributions and percentages (A), measured by partial least squares discrimination analysis (PLS‐DA), and the carbon atom map (B) in healthy controls (red) and patients with ADC (green), SCLC (orange), and SCC (blue). Each of selected five principal components represents as the model to interpret that values of abscissa and ordinate represents the distance from the sample nodule to the origin of the center after projecting to a plane in multidimensional space (A). The atom map describes the expression of major carbon atom number between 14 and 22 in various lipid types (B)

Total 502 lipid elements of plasma were identified qualitatively and quantitatively, mainly including 78 PAs, 60 PCs, 53 PEs, 52 PGs, 52 PIs, 57 PSs, 21 lysophosphatidylcholines (lysoPC), 17 lysophosphatidylethanolamines (lysoPE), 21 lysophosphatidylglycerols (lysoPG), 21 lysophosphatidylinositols (lysoPI), 21 lysophosphatidylserines (lysoPS), nine diacylglycerides, and 11 tri‐acylglycerols (TAG). Levels of some lipid elements in ADC, SCC, or SCLC patients were significantly higher (Table S2) or lower (Table S3), as compared with healthy control (> twofold; *P* < .05). The majority of those elevated lipid elements were PC (36%), PA (30%), and lysoPC (10%) in ADC; PE (26%), PC (20%), PS (17%), and PG (16%) in SCC; or PS (16%), PE (14%), PG (14%), lysoPS (17%), and lysoPI (16%) in SCLC. Of those declined lipid elements, 72% were PS in ADC, whereas 81% and 94% were PA in SCC and SCLC, respectively. Table 3 demonstrates lung cancer subtype‐specific lipid elements identified by those lipid elements elevated or declined exclusively in each lung cancer subtype, for example, some of lysoPC and PC in ADC, whereas lysoPI, lysoPS, PE, and PA in SCLC. By partial least squares discrimination analysis (PLS‐DA) analysis, top 15 lipid elements were defined on the basis of variable import in project (VIP) score of each group: TAG565, lysoPG182, and PG (344, 320, 321, 300, and 341) increased in ADC; lysoPG181, PA140/245, PI384/180, PA (140/205 and 180/205), and PE385/PE180 increased in SCLC; and PI362/PI180 and lysoPG182 decreased in SCC (detail in Figure 1A). There was a clear distribution of top 25 lipid elements among lung cancer groups, as compared with the healthy control (Figure 1B). Of those significantly increased lipid elements in patients with lung cancers, top six lipids of each group were identified (Figure 2): levels of LysoPC (16:0 sn‐2, 17:0/1 sn‐1, and 18:0/1 sn‐1) in ADC (Figure 2A), PS36:3 in SCC (Figure 2B), and PA (14:0/24:5 and 14:0/20:5) and PI (40:1, 18:1, and 22:0) in SCLC (Figure 2C) were significantly higher than in other three groups. PLS‐DA component analysis demonstrated that five principal components selected were 32.6%, 39.6%, 13.3%, 3.1%, and 4% (Figure 3A). In the atom map, which was based on the expression of major C atom numbers in various lipid types, levels of lipids with carbons (14:0, 20:1, and 22:6) increased, whereas those with carbons (15:0, 18:0, 19:0, 20:5) decreased, as compared with healthy control (Figure 3B).

**TABLE 3:** Lung cancer subtype‐associated lipid elements significantly elevated or declined alone in patients with adenocarcinoma, squamous cell carcinoma, or small cell lung cancer (more than twofold) as compared with healthy control (*P*‐values)

Adenocarcinoma			Squamous cell carcinoma			Small cell lung cancer		
Lipids	Folds	*P*‐values	Lipids	Folds	*P*‐values	Lipids	Folds	*P*‐values
**Elevated > twofold**								
lysoPC 16:0 (sn‐2)	11.81	.01	d181So	11.88	.05	lysoPG 14:0	6.02	.00
lysoPC 17:0 (sn‐1)	11.33	.01	C1P240	7.46	.05	lysoPI 15:1 (sn‐1)	2.65	.02
lysoPC 17:1 (sn‐1)	6.92	.02	d18:1S1P	6.11	.04	lysoPI 16:0 (sn‐2)	5.38	.02
lysoPC 18:0 (sn‐1)	6.45	.02	PS36:3	2.39	.04	lysoPI 17:0 (sn‐1)	4.47	.00
lysoPC 18:1 (sn‐1)	6.40	.00				lysoPI 18:0 (sn‐1)	6.40	.02
lysoPC 20:0 (sn‐1)	5.82	.00				lysoPI 18:1 (sn‐1)	5.47	.01
lysoPC 20:1 (sn‐1)	5.79	.00				lysoPI 18:2 (sn‐1)	5.41	.04
lysoPC 20:4 (sn‐1)	5.50	.02				lysoPI 18:3 (sn‐1)	3.51	.03
lysoPC 22:0 (sn‐1)	5.21	.00				lysoPI 19:0 (sn‐1)	7.29	.00
lysoPC 22:6 (sn‐1)	5.16	.00				lysoPI 20:0 (sn‐1)	5.76	.00
lysoPE 19:0	5.14	.03				lysoPI 20:2 (sn‐1)	7.60	.00
lysoPG 16:0	4.66	.01				lysoPI 20:4 (sn‐1)	3.91	.01
lysoPG 16:1	4.49	.02				lysoPS 15:0	3.07	.00
lysoPS 16:0	4.26	.01				lysoPS 16:1	16.30	.02
lysoPS 18:2	4.23	.01				lysoPS 18:1	3.37	.02
lysoPS 20:0	3.98	.02				lysoPS 19:0	4.37	.01
PA 18:1/20:4	3.45	.02				lysoPS 20:1	3.05	.01
PA 18:2/20:4	3.45	.04				lysoPS 20:2	4.73	.02
PA 18:4/19:0	3.30	.02				lysoPS 20:4	3.22	.05
PC 19:0/19:0	3.08	.01				lysoPS 20:5	3.80	.01
PC 19:0/21:2	3.05	.02				lysoPS 22:4	4.85	.02
PC 33:2e; PC 16:1e/18:1	3.02	.02				PA 14:0/20:5	20.60	.03
PC 35:2e; PC 16:0e/20:2	2.90	.00				PA 14:0/24:5	26.30	.04
PC 35:3; PC 17:1/18:2	2.88	.02				PE 36:2; PE 18:0/18:2 or 18:1/18:1	3.00	.04
PC 36:5; PC 14:0/22:5 or 16:0/20:5; 16:1/20:4	2.74	.02				PE 36:4; PE 16:0/20:4	3.32	.04
PC 38:2; PC 16:0/22:2	2.51	.01				PI 39:7; PI 17:1/22:6	2.05	.04
PC 38:7; PC 16:1/22:6 or 18:2/20:5	2.41	.02				PI 40:1; PI 18:1/22:0	15.70	.01
PE 36:6; PE 16:1/20:5	2.06	.02				PI 41:6; PI 19:0/22:6	2.76	.03
						PIP 36:1	3.15	.01
						PS 32:1	5.53	.01
						PS 37:2	2.47	.02
						TAG 56:5	3.56	.01
**Declined > twofold**								
PG 37:6	0.49	.05	PG 18:0/19:0	0.44	.03	PA 10:0/18:3	0.31	.04
PS 33:0	0.39	.01	PS40:1	0.30	.04	PA 15:0/20:2	0.29	.04
PS 37:5	0.20	.00				PA 16:0/18:4	0.17	.03
SM240	0.05	.00				PA 18:0/18:4	0.39	.02
						PA 18:1/ 18:4	0.49	.02
						PA 18:1/18:4	0.47	.02
						PA 18:1/22:4	0.46	.02
						PA 18:3/20:0	0.37	.02
						PA 18:4/20:1	0.43	.02

**FIGURE 4:**
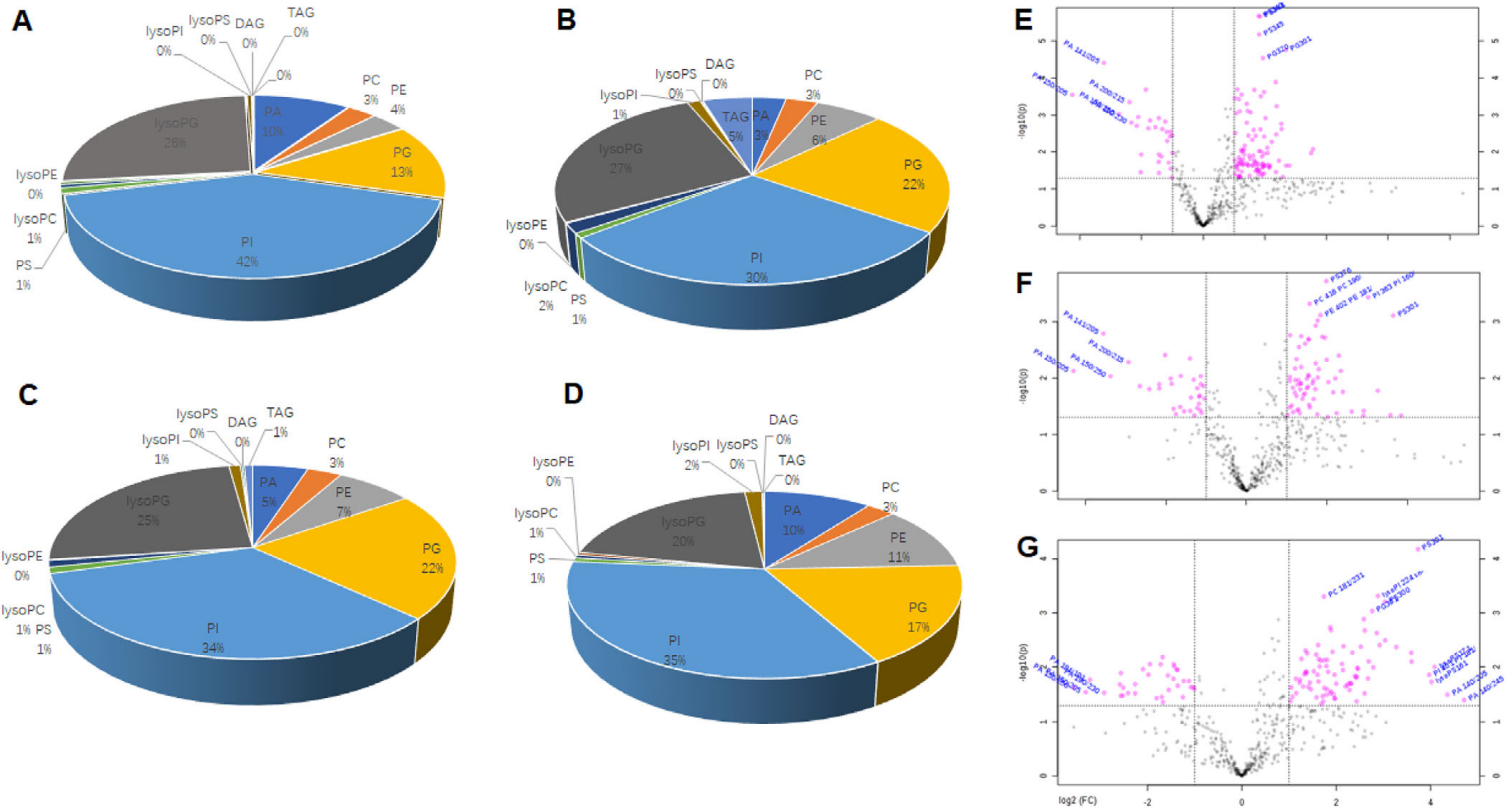
The proportion (%) of 13 main lipid elements of healthy controls (A), ADC (B), SCC (C), and SCLC (D) and volcanic map between heathy controls with ADC (E), SCC (F), or SCLC (G), respectively. The lipid elements were identified on the basis of statistical significance. The abscissa represents log values of fold changes, where the left side of the first dotted line perpendicular to the abscissa represents <0.5‐fold changes and the right side of the second dotted line represents >2‐fold changes. The vertical coordinate represents –log10 (*P*‐value). The upper side of the dotted line perpendicular to the ordinate stands for *P*‐value less than .05, as compared with healthy controls

As compared with the healthy control (Figure 4A), we noticed that PI mainly declined in ADC (Figure 4B), PA in ADC and SCC (Figure 4C), and lysoPG in SCLC (Figure 4D), whereas PG and TAG increased in ADC and SCC, PE in SCLC, and PC in SCC. The volcanic map demonstrated the clear patterns of lipid elements significantly increased or declined between heathy controls with ADC (Figure 4E), SCC (Figure 4F), or SCLC (Figure 4G) and varied among different subtypes of lung cancers.

### 3.3 Different lipidomics between patient genders

About 81 or 79 lipid elements significantly increased and 38 or 21 declined more than twofold in male or female lung cancer patients, as compared with male or female healthy controls, respectively (Tables S4 and S5). Of those, PC and PE mainly elevated in male and female patients, whereas PA declined in both, although the number of PA in male patients was more than in female patients. Table 4 demonstrates gender‐specific lipid elements identified by those lipid elements elevated or declined exclusively in either male or female lung cancer patients, for example, some of lysoPS, PC, and PS elevated in male patients, whereas lysoPI and PE in female patients. There were about 45 or 28 increased or declined lipid elements differed between male and female lung cancer patients (Table 4).

**TABLE 4:** Gender‐associated lipid elements significantly elevated or declined alone in male or female patients with lung cancer (more than twofold) as compared with healthy control (*P*‐values)

Male patients with lung cancer			Female patients with lung cancer		
Lipids	Folds	*P*‐values	Lipids	Folds	*P*‐values
**Elevated > twofold**					
lysoPI 20:3 (sn‐1)	4.82	.03	C1P120 Mean	4.27	.03
lysoPS 16:0	5.07	.01	C1P160 Mean	8.00	.03
lysoPS 17:0	3.64	.01	C1P240 Mean	11.50	.01
lysoPS 17:1	12.47	.04	Cer120	4.29	.04
lysoPS 18:1	3.77	.03	d171So	4.19	.04
lysoPS 18:3	6.24	.01	d18:0Sa1P	3.64	.04
lysoPS 20:1	2.69	.02	d18:1S1P	4.82	.05
lysoPS 20:2	3.80	.03	d181So	6.86	.02
PC 37:3; PC 17:0/20:3 or 19:1/18:2	2.07	.03	lysoPC 22:6 (sn‐1)	2.67	.04
PC 37:4; PC 17:0/20:4	2.09	.01	lysoPI 18:0 (sn‐1)	2.80	.01
PC 37:5e;PC 16:0e/22:5;18:0e/20:5	2.03	.01	lysoPI 18:1 (sn‐1)	3.48	.02
PC 39:2 (18:0/21:2)	2.62	.04	lysoPI 20:2 (sn‐1)	5.16	.04
PC 39:3 (18:0/21:3)	3.44	.03	lysoPI 22:6 (sn‐1)	7.00	.00
PC 39:3; PC 19:0/20:3	3.43	.03	lysoPS 15:1	3.64	.02
PC 39:4 (18:0/21:4)	3.30	.02	PC 16:0/26:0	2.42	.03
PC 39:5 (18:0/21:5)	2.93	.02	PC 18:1/23:1	6.85	.01
PC 39:6; PC 17:0/22:6	3.09	.01	PE 35:5p; PE 16:0p/20:4	2.24	.02
PC 40:1; PC 18:1/22:0	2.47	.04	PE 35:6p; PE 16:0p/20:5	2.49	.02
PC 41:6; PC 19:0/22:6	4.81	.04	PE 36:5; PE 16:0/20:5	2.08	.04
PC 42:5	2.89	.01	PE 37:6p; PE 18:0p/20:5 or 18:1p/20:4; 16:0e/22:6	2.34	.02
PE 40:7; PE 18:1/22:6	3.81	.03	PE 37:7p; PE 16:0p/22:6	2.56	.01
PG 36:1	2.38	.01	PE 38:5; PE 18:0/20:5	2.22	.04
PG 36:2	2.27	.02	PE 38:7; PE 16:1/22:6 or 18:2/20:5	2.56	.03
PI 31:1p; PI 16:0p/16:0	2.39	.04	PI 36:3; PI 16:0/20:3 or 18:0/18:3 or 18:1/18:2	8.33	.00
PS 32:0	3.41	.02	PS 35:5	2.32	.03
PS 32:1	6.28	.02	PS 37:2	2.23	.03
PS 33:2	2.23	.01			
PS 35:3	3.11	.00			
PS 37:3	2.97	.01			
PS 37:5	2.12	0.01			
**Declined > twofold**					
lysoPS 20:0	0.45	.04	PA 16:0/18:4	0.25	.01
PA 10:0/18:1	0.40	.00	PA 16:1/18:4	0.32	.02
PA 15:0/20:2	0.47	.01			
PA 16:0/22:4	0.44	.00			
PA 17:0/13:0	0.37	.00			
PA 18:1/20:4	0.25	.00			
PA 18:2/20:4	0.26	.02			
PA 18:3/20:4	0.47	.00			
PA 18:4/20:2	0.39	.01			
PA 20:4/26:2	0.40	.00			
PG 18:0/19:0	0.42	.00			
PG 37:1	0.39	.01			
PS 38:1	0.46	.00			
PS 40:1	0.36	.01			
PS 41:6	0.45	.00			

### 3.4 Different lipidomics among patient ages, stags, metastases, and survival status

About 106, 88, and 82 lipid elements significantly elevated or 47, 44, and 18 declined in lung cancer patients at <60, 60‐70, >70 years old, respectively, as compared with healthy controls (*P* < .05). We noticed that elements of PG and PS mainly increased in lung cancer patients at all age groups: for example, 19% and 18% at <60‐year group; 17% and 20% at 60‐ to 70‐year group; and 16% and 16% at >70‐year group; lysoPC and PC increased at <60‐year group (18% and 16%) and at >70‐year group (16% and 24%); PE increased at 60‐ to 70‐year group (17%) and at >70‐year group (22%), as detailed in Table S6. Elements of PA mainly declined in lung cancer patients at all ages (Table S7). Of those significantly altered lipid elements, 13%, 20%, and 15% appeared only at <60‐year, 60‐ to 70‐year, and >70‐year groups, respectively, and considered as age‐specific lipid elements (Table 5). LysoPC and lysoPI mainly increased in <60‐year and 60‐ to 70‐year old patients, whereas lysoPE declined in <60‐year group. We also compared lipidomic profiles between patients at early and late stages of lung cancer, and found 72 and 86 lipid elements significantly increased at early and late stages, respectively, of which PE, PG, ad PS increased in both stages, lysoPI in early stage, and PC in late stage (Table S8). About 10 and 42 elements declined at early and late stages, where the majority was PA (Table S9). Table 6 demonstrates stage‐specific lipid elements identified by those lipid elements elevated or declined exclusively at early and late stages of lung cancer, for example, some of lysoPI and PE elevated at early stage, and lysoPC and PE at late stage.

**TABLE 5:** Age‐associated lipid elements significantly elevated or declined alone in each age group of patients with lung cancer (more than twofold) as compared with healthy control (*P*‐values)

Patient age <60			Patient age = 60‐70			Patient age >70		
Lipids	Folds	*P*‐values	Lipids	Folds	*P*‐values	Lipids	Folds	*P*‐values
**Elevated > twofold**								
C1P120 Mean	20.07	.04	lysoPG14:0	6.28	.00	d18:1S1P	7.97	.04
lysoPC 15:0 (sn‐2)	3.11	.03	lysoPI 16:0 (sn‐2)	3.89	.04	lysoPC 22:4 (sn‐1)	2.24	.03
lysoPC 15:1 (sn‐1)	6.94	.02	lysoPI 17:0 (sn‐1)	3.16	.02	lysoPE19:0	7.21	.01
lysoPC 16:1 (sn‐1)	3.67	.02	lysoPI 18:1 (sn‐1)	4.42	.02	lysoPE19:1	3.30	.05
lysoPC 17:0 (sn‐1)	3.53	.01	lysoPI 18:3 (sn‐1)	2.90	.04	PC 16:0/26:0	3.82	.00
lysoPC 18:2 (sn‐1)	5.61	.05	lysoPI 19:0 (sn‐1)	6.62	.00	PC 19:0/19:0	2.99	.00
lysoPC 18:3 (sn‐1)	4.93	.02	lysoPI 20:0 (sn‐1)	4.36	.01	PC 19:0/21:2	3.78	.00
lysoPC 20:2 (sn‐1)	3.29	.05	lysoPI 20:3 (sn‐1)	5.34	.01	PE 35:6p; PE16:0p/20:5	2.22	.02
lysoPG 15:1	2.74	.04	lysoPI 20:4 (sn‐1)	2.99	.03	PE 37:3; PE 17:0/20:3	2.53	.02
lysoPG 16:0	3.01	.02	lysoPI 22:4 (sn‐1)	6.56	.00	PE 38:7; PE 16:1/22:6 or 18:2/20:5	2.31	.02
lysoPS 15:1	5.27	.00	lysoPS15:0	2.92	.01	PE 38:7; PE 16:1/22:6 or 18:2/20:5	2.79	.02
lysoPS 18:1	3.72	.05	lysoPS16:1	9.51	.01	PE 42:8; PE 20:2/22:6	3.13	.03
lysoPS 19:0	5.66	.04	lysoPS17:0	3.83	.02	PG35:1	2.27	.02
lysoPS 20:3	2.33	.02	lysoPS20:1	3.01	.01	PS35:4	2.62	.04
PC 39:2 (18:0/21:2)	3.06	.03	lysoPS20:2	4.61	.02	PS37:2	2.64	.02
PE 38:5; PE 18:0/20:5	2.38	.05	lysoPS22:0	3.77	.01			
PG 33:2	2.11	.01	PA 14:0/20:5	13.29	.05			
PG 35:2	2.28	.03	PE 36:2; PE 18:0/18:2 or 18:1/18:1	2.51	.04			
PG 36:6	2.54	.01	PE 36:4; PE 16:0/20:4	2.76	.04			
PS 35:1	2.30	.00	PE 38:4; PE 16:0/22:4	3.26	.03			
			PE 38:6; PE 16:0/22:6 or 16:1/22:5;20:2/18:4	3.69	.02			
			PI 31:1p; PI16:0p/16:0	2.04	.02			
			PI 40:1; PI 18:1/22:0	11.56	.03			
			PI 41:6; PI 19:0/22:6	2.26	.04			
			PI36:1	2.40	.04			
			TAG 53:2	4.00	.00			
**Declined > twofold**								
lysoPE 16:0 (sn‐1)	0.41	.04	PA 11:0/22:6	0.49	.02	PA 18:0/20:5	0.32	.02
lysoPE 18:0 (sn‐1)	0.40	.04	PA 17:0/13:0	0.30	.01			
lysoPE 18:1 (sn‐1)	0.47	.04	PG39:3	0.41	.02			
lysoPE 18:2 (sn‐2)	0.40	.04						
PA 16:1/18:4	0.30	.02						
PA 18:1/20:4	0.22	.04						
PS 38:1	0.44	.02						
PS 40:1	0.27	.02						

**TABLE 6:** Stage‐associated lipid elements significantly elevated or declined alone in patients with lung cancer at the early stage or late stage (more than two folds), respectively, as compared with healthy control (*P*‐values)

Patient at early stage			Patients at late stage			Patients at late stage		
Lipids	Folds	*P*‐values	Lipids	Folds	*P*‐values	Lipids	Folds	*P*‐values
**Elevated > twofold**						**Declined > twofold**		
lysoPE 19:0	6.36	.01	lysoPC 17:0 (sn‐1)	2.61	.04	lysoPS 20:0	0.38	.01
lysoPG 18:3	2.18	.02	lysoPC 18:0 (sn‐1)	2.68	.03	PA 10:0/18:1	0.36	.00
lysoPI 17:0 (sn‐1)	7.39	.04	lysoPC 19:0 (sn‐1)	2.79	.01	PA 10:0/18:2	0.36	.00
lysoPI 18:0 (sn‐1)	3.90	.01	lysoPC 20:1 (sn‐1)	2.10	.05	PA 10:0/18:3	0.48	.01
lysoPI 18:1 (sn‐1)	4.44	.01	lysoPC 22:6 (sn‐1)	2.91	.05	PA 15:0/18:0	0.43	.00
lysoPI 18:2 (sn‐1)	3.06	.04	lysoPG 15:1	2.19	.05	PA 15:0/18:2	0.49	.00
lysoPI 18:3 (sn‐1)	2.95	.03	lysoPS 18:1	3.36	.04	PA 15:0/20:2	0.43	.00
lysoPI 19:0 (sn‐1)	13.11	.00	lysoPS 18:3	4.89	.01	PA 15:1/24:0	0.26	.00
lysoPI 20:0 (sn‐1)	5.71	.00	lysoPS 20:2	3.30	.04	PA 16:0/20:1	0.44	.00
lysoPI 20:1 (sn‐1)	3.77	.01	lysoPS 22:6	3.37	.04	PA 16:0/22:4	0.43	.00
lysoPI 20:2 (sn‐1)	11.62	.00	PC 37:3; PC 17:0/20:3 or 19:1/18:2	2.09	.03	PA 17:0/13:0	0.43	.00
lysoPI 20:3 (sn‐1)	6.60	.01	PC 37:4; PC 17:0/20:4	2.17	.02	PA 17:0/20:5	0.31	.00
lysoPI 20:4 (sn‐1)	3.55	.02	PC 37:5e; PC 16:0e/22:5 or 18:0e/20:5	2.07	.01	PA 18:0/20:1	0.44	.00
lysoPI 22:4 (sn‐1)	4.75	.00	PC 39:2 (18:0/21:2)	2.53	.05	PA 18:0/22:4	0.37	.00
lysoPI 22:6 (sn‐1)	8.18	.00	PC 39:3 (18:0/21:3)	3.52	.03	PA 18:1/20:4	0.25	.00
lysoPS 15:0	2.54	.02	PC 39:3; PC 19:0/20:3	3.53	.03	PA 18:2/18:4	0.49	.00
lysoPS 15:1	3.71	.01	PC 39:4 (18:0/21:4)	3.53	.03	PA 18:2/20:4	0.26	.01
lysoPS 20:3	2.08	.01	PC 39:5 (18:0/21:5)	3.10	.03	PA 18:3/18:4	0.28	.00
lysoPS 22:0	2.98	.00	PC 39:6; PC 17:0/22:6	3.18	.01	PA 18:3/20:4	0.44	.00
PC 19:0/19:0	2.32	.03	PC 39:7; PC 17:1/22:6	2.72	.00	PA 18:3/21:0	0.45	.01
PC 19:0/21:2	2.46	.04	PC 40:1; PC 18:1/22:0	2.47	.03	PA 18:3/22:4	0.36	.00
PE 32:2; PE 14:0/18:2	3.42	.03	PC 40:4; PC 18:0/22:4 or 20:1/20:3	2.44	.00	PA 18:4/19:0	0.30	.00
PE 35:2; PE 17:0/18:2	2.37	.02	PC 41:6; PC 19:0/22:6	4.93	.03	PA 18:4/20:2	0.34	.00
PE 35:6p; PE 16:0p/20:5	2.27	.02	PC 42:5	2.87	.01	PA 18:4/20:4	0.44	.00
PE 36:5; PE 16:0/20:5	2.05	.04	PE 35:5p; PE 16:0p/20:4	2.47	.03	PA 18:4/20:5	0.45	.00
PE 37:3; PE 17:0/20:3	2.55	.03	PE 37:6p; PE 18:0p/20:5 or 18:1p/20:4 or 16:0e/22:6	2.66	.03	PA 19:0/22:5	0.14	.00
PE 40:1; PE 22:0/18:1	2.71	.00	PE 37:7p; PE 16:0p/22:6	2.89	.03	PA 19:0/23:0	0.13	.00
PI 30:1	2.03	.04	PE 38:3; PE 18:0/20:3	3.33	.01	PA 20:4/26:2	0.41	.00
PI 31:1p; PI 16:0p/16:0	2.19	.02	PE 40:4; PE 18:0/22:4 or 20:0/20:4	3.24	.01	PG 18:0/19:0	0.34	.00
PI 36:3; PI 16:0/20:3 or 18:0/18:3 or 18:1/18:2	8.01	.00	PE 40:7; PE 18:1/22:6	3.85	.02	PG 37:1	0.46	.02
PS 33:0	2.26	.01	PE 42:8; PE 20:2/22:6	4.29	.05	PS 40:1	0.37	.00
PS 35:5	2.62	.03	PG 32:1	3.05	.00	PS 41:6	0.47	.00
			PG 33:0	2.69	.00			
			PG 33:1	2.64	.00			
			PG 34:0	3.99	.00			
			PG 34:1	4.07	.00			
			PG 34:2	2.19	.00			
			PG 35:4	2.00	.03			
			PG 36:1	2.15	.01			
			PG 36:2	2.05	.02			
			PS 32:0	3.20	.02			
			PS 37:2	3.68	.05			
			PS 38:7	2.98	.02			

We noticed about 54 or 89 lipid elements significantly increased in patients without or with metastasis, of which lysoPI mainly elevated in patients without metastasis, whereas PC and PE in patients with metastasis (Table S10). The declined number of lipid elements, especially PA in patients with metastasis, was significantly higher than in patients without metastasis (Table S11). There were about 58 or 25 elevated or declined lipid elements in patients with metastasis, of which PA was majority of declined elements in patients with metastasis (Table 7).

**TABLE 7:** Metastasis‐associated lipid elements significantly elevated or declined alone in lung cancer patients with metastasis (more than twofold), as compared with healthy control (*P*‐values)

Elevated > twofold			Declined > twofold		
Lipid elements	Folds	*P*‐values	Lipid elements	Folds	*P*‐values
lysoPC 17:0 (sn‐1)	2.96	.03	lysoPS 20:0	0.39	.02
lysoPC 18:0 (sn‐1)	2.97	.02	PA 10:0/18:1	0.39	.00
lysoPC 19:0 (sn‐1)	2.99	.01	PA 15:0/18:0	0.45	.00
lysoPC 20:0 (sn‐1)	2.55	.02	PA 15:0/18:2	0.49	.00
lysoPC 22:0 (sn‐1)	3.03	.02	PA 15:0/20:2	0.46	.01
lysoPC 22:6 (sn‐1)	3.20	.04	PA 16:0/20:1	0.43	.00
lysoPG 15:1	2.27	.04	PA 16:0/22:4	0.46	.00
lysoPI 22:0 (sn‐1)	11.39	.01	PA 17:0/13:0	0.41	.00
lysoPS 15:0	2.46	.04	PA 17:0/20:5	0.33	.00
lysoPS 16:0	3.68	.02	PA 18:0/20:1	0.46	.00
lysoPS 17:1	11.04	.04	PA 18:0/22:4	0.40	.00
lysoPS 18:1	3.25	.04	PA 18:1/20:4	0.26	.00
lysoPS 18:2	5.21	.01	PA 18:2/20:4	0.26	.01
lysoPS 22:0	2.74	.04	PA 18:3/20:4	0.46	.00
lysoPS 22:6	2.97	.03	PA 18:3/21:0	0.46	.01
PC 37:3; PC 17:0/20:3 or 19:1/18:2	2.25	.02	PA 18:3/22:4	0.39	.00
PC 37:4; PC 17:0/20:4	2.30	.02	PA 18:4/19:0	0.31	.00
PC 37:5e; PC 16:0e/22:5 or 18:0e/20:5	2.21	.01	PA 18:4/20:2	0.38	.00
PC 39:2 (18:0/21:2)	2.82	.04	PA 18:4/20:4	0.47	.00
PC 39:3 (18:0/21:3)	3.88	.03	PA 18:4/20:5	0.44	.00
PC 39:4 (18:0/21:4)	3.89	.03	PA 19:0/22:5	0.14	.00
PC 39:5 (18:0/21:5)	3.37	.03	PG 18:0/19:0	0.34	.00
PC 39:6; PC 17:0/22:6	3.48	.01	PG 37:1	0.48	.04
PC 39:7; PC 17:1/22:6	2.95	.00	PS 40:1	0.40	.01
PC 40:1; PC 18:1/22:0	2.74	.02	PS 41:6	0.42	.00
PC 40:4; PC 18:0/22:4 or 20:1/20:3	2.64	.00			
PC 42:5	3.19	.01			
PE 35:5p; PE 16:0p/20:4	2.57	.02			
PE 35:6p; PE 16:0p/20:5	2.60	.04			
PE 36:1; PE 16:0/20:1 or 18:0/18:1	2.74	.01			
PE 37:6p; PE 18:0p/20:5 or 18:1p/20:4 or 16:0e/22:6	2.83	.03			
PE 37:7p; PE 16:0p/22:6	3.06	.03			
PE 38:2; PE 18:1/20:1	3.91	.01			
PE 38:3; PE 18:0/20:3	3.37	.01			
PE 38:6; PE 16:0/22:6 or 16:1/22:5 or 20:2/18:4	2.76	.05			
PE 40:3; PE 18:1/22:2 or 22:1/18:2	5.03	.00			
PE 40:4; PE 18:0/22:4 or 20:0/20:4	3.32	.01			
PE 40:7; PE 18:1/22:6	4.14	.01			
PE 42:8; PE 20:2/22:6	4.64	.04			
PG 30:0	5.06	.00			
PG 32:0	3.66	.00			
PG 32:1	3.06	.00			
PG 32:2	3.13	.00			
PG 33:0	2.73	.00			
PG 33:1	2.65	.00			
PG 34:0	3.96	.00			
PG 34:1	4.06	.00			
PG 34:2	2.14	.00			
PG 35:4	2.06	.02			
PG 36:2	2.24	.02			
PS 30:0	6.53	.00			
PS 32:0	3.23	.02			
PS 33:2	2.13	.01			
PS 34:2	2.68	.01			
PS 35:1	2.08	.01			
PS 35:3	2.79	.00			
PS 37:2	3.16	.04			
PS 38:7	3.38	.02			

Lipidomic panel also differed between survived and nonsurvived patients. There were only eight lipids exclusively elevated in nonsurvived patients, that is, lysoPS14:0, PC 39:3 (18:0/21:3), PC 39:5 (18:0/21:5), PE 38:7; PE 16:1/22:6 or 18:2/20:5, PE 40:7; PE 18:1/22:6, PS33:0, PS37:2, and PS38:7. However, far more lipids^31^ elevated alone in survived patients, mainly elements of lysoPC, lysoPG, lysoPI, lysoPS, and PS. On the contrary, there were no lipids declined alone in survived patients, while 19 lipids in non‐survived patients, 17 of which were PA elements (Table 8).

**TABLE 8:** Lipid elements significantly elevated or declined alone in lung cancer patients survived or nonsurvived (more than twofold), respectively, as compared with healthy control (*P*‐values)

Survived			Nonsurvived		
Lipid	Folds	*P*‐values	Lipid	Folds	*P*‐values
**Elevated**					
C1P120 Mean	3.21	.04	lysoPS14:0	4.50	.03
C1P240 Mean	10.16	.00	PC 39:3 (18:0/21:3)	2.92	.04
d181So	6.19	.05	PC 39:5 (18:0/21:5)	2.48	.05
lysoPC 15:1 (sn‐1)	2.62	.04	PE 38:7; PE 16:1/22:6 or 18:2/20:5	2.17	.03
lysoPC 20:0 (sn‐1)	2.45	.03	PE 40:7; PE 18:1/22:6	2.23	.04
lysoPC 22:0 (sn‐1)	3.28	.02	PS 33:0	2.37	.04
lysoPE 19:0	5.54	.04	PS 37:2	4.94	.04
lysoPG 14:0	5.75	.00	PS 38:7	3.25	.02
lysoPG 16:1	3.14	.01			
lysoPG 18:3	2.03	.04			
lysoPI 20:0 (sn‐1)	4.10	.04			
lysoPI 20:2 (sn‐1)	6.86	.03			
lysoPI 22:0 (sn‐1)	9.03	.01			
lysoPI 22:6 (sn‐1)	6.96	.02			
lysoPS 15:1	3.64	.03			
lysoPS 16:0	2.21	.02			
lysoPS 17:0	2.49	.03			
lysoPS 19:0	4.25	.03			
lysoPS 20:5	3.23	.01			
lysoPS 22:4	2.44	.03			
PC 39:3; PC 19:0/20:3	2.10	.03			
PC 41:6; PC 19:0/22:6	2.45	.01			
PE 35:5p; PE 16:0p/20:4	2.30	.04			
PE 35:6p; PE 16:0p/20:5	2.45	.03			
PI 36:3; PI 16:0/20:3 or 18:0/18:3 or 18:1/18:2	10.38	.00			
PS 30:0	4.75	.00			
PS 32:1	3.32	.03			
PS 33:2	2.25	.02			
PS 35:1	2.26	.00			
PS 35:4	2.74	.02			
PS 37:3	2.47	.00			
PS 37:5	2.00	.04			
SM d18:0/26:1 +HN4+	4.95	.00			
SM120	2.16	.03			
TAG 53:2	3.74	.02			
**Declined**					
			PA 10:0/18:2	0.34	.00
			PA 14:1/21:4	0.24	.00
			PA 15:0/25:0	0.12	.00
			PA 15:1/18:1	0.49	.01
			PA 15:1/24:0	0.25	.00
			PA 16:0/20:1	0.44	.01
			PA 16:1/18:4	0.33	.02
			PA 17:0/20:5	0.25	.01
			PA 18:0/20:1	0.42	.00
			PA 18:0/22:4	0.42	.01
			PA 18:1/20:4	0.31	.04
			PA 18:3/18:4	0.29	.00
			PA 18:4/19:0	0.25	.014
			PA 18:4/20:2	0.35	.02
			PA 18:4/20:5	0.44	.00
			PA 19:0/22:5	0.18	.00
			PA 19:0/23:0	0.16	.00
			PG 18:0/19:0	0.38	.00
			PG 40:6	0.44	.00

### 3.5 Trans‐omic profiles between clinical phenomes and lipidomes

**FIGURE 5:**
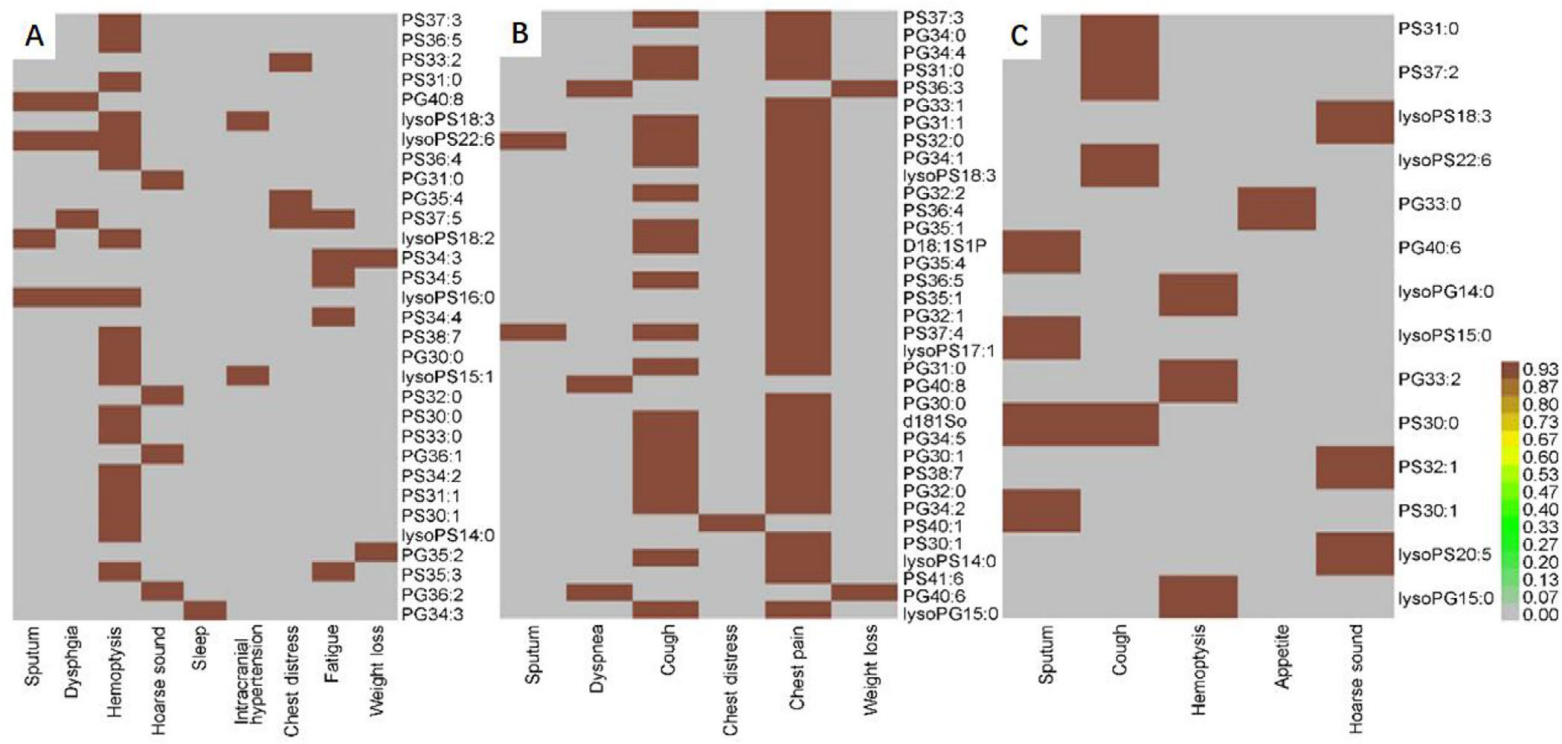
Trans‐omic nodules cross‐clinical phenomes and lipidomes measured by simulating the expression quantitative trait locus (eQTL) model. The abscissa represents the related clinical phenomes, and the ordinate represents the corresponding lipid elements with *P*‐values less than .05 in patients with ADC (A), SCC (B), and SCLC (C), as compared with healthy controls

We also compared the difference of lipidomic profiles between general metabolism statuses of patients indicated by BMI and between degrees of clinical phenomes measured by DESS scores. Levels of lysoPC or lysoPI mainly elevated in patients with BMI ≤ 22 or > 22, respectively (Table S12), whereas the number of declined PA in patients with BMI ≤ 22 was higher than that in patients with BMI > 22 (Table S13). About 42 BMI‐associated lipid elements significantly elevated or declined exclusively in patients with BMI ≤ 22, and about 22 in patients with BMI > 22 (Table 9). Levels of lysoPC and PE or PG and PS mainly increased in patients with DESS ≤ 90 or > 90 (Table S14). The number of declined PA (n = 19) in patients with DESS ≤ 90 was lower than that in patients with DESS > 90 (n = 44). Figure 5 demonstrates the variation of trans‐omic profiles among lung cancer subtypes indicated by trans‐omic nodules cross significant networks of clinical phenome and lipidome layers.

**TABLE 9:** BMI‐associated lipid elements significantly elevated or declined alone in lung cancer patients with BMI < 22 or > 22 (more than twofold), respectively, as compared with healthy control (*P*‐values)

Patient BMI < 22			Patients BMI > 22		
Lipid elements	Folds	*P*‐values	Lipid elements	Folds	*P*‐values
Elevated > twofold					
lysoPC 14:0 (sn‐2)	3.46	.04	lysoPI 18:0 (sn‐1)	4.99	.03
lysoPC 15:0 (sn‐2)	2.77	.05	lysoPI 18:1 (sn‐1)	5.48	.03
lysoPC 15:1 (sn‐1)	4.72	.04	lysoPI 19:0 (sn‐1)	7.22	.03
lysoPC 16:0 (sn‐2)	2.75	.03	lysoPI 20:0 (sn‐1)	3.89	.02
lysoPC 16:1 (sn‐1)	3.99	.02	lysoPI 20:2 (sn‐1)	7.44	.03
lysoPC 17:0 (sn‐1)	2.88	.02	lysoPI 20:3 (sn‐1)	5.11	.01
lysoPC 17:1 (sn‐1)	2.91	.03	lysoPI 20:4 (sn‐1)	2.85	.04
lysoPC 18:0 (sn‐1)	2.86	.01	lysoPI 22:4 (sn‐1)	5.53	.00
lysoPC 18:1 (sn‐1)	2.89	.03	lysoPI 22:6 (sn‐1)	6.81	.01
lysoPC 18:3 (sn‐1)	4.40	.03	lysoPS 15:1	4.46	.01
lysoPC 19:0 (sn‐1)	3.18	.00	lysoPS 17:0	3.07	.02
lysoPC 20:0 (sn‐1)	2.71	.01	lysoPS 20:1	2.28	.03
lysoPC 20:1 (sn‐1)	2.25	.04	lysoPS 20:2	2.74	.04
lysoPC 20:2 (sn‐1)	2.37	.02	lysoPS 20:5	2.88	.02
lysoPC 20:3 (sn‐1)	2.61	.02	PE 40:1; PE 22:0/18:1	2.50	.01
lysoPC 20:4 (sn‐1)	3.12	.03	PI 30:1	2.13	.01
lysoPC 22:6 (sn‐1)	2.95	.02	PI 31:1p; PI 16:0p/16:0	2.36	.00
lysoPS 15:0	2.44	.03	PI 36:3; PI 16:0/20:3; 18:0/18:3; 18:1/18:2	11.12	.00
lysoPS 18:1	2.99	.04			
lysoPS 22:4	2.45	.02			
lysoPS 22:6	3.05	.02			
PC 18:1/23:1	11.58	.05			
PC 37:3; PC 17:0/20:3 or 19:1/18:2	2.23	.03			
PC 39:3 (18:0/21:3)	3.80	.03			
PC 39:3; PC 19:0/20:3	3.81	.03			
PC 39:4 (18:0/21:4)	4.02	.03			
PC 39:5 (18:0/21:5)	3.44	.03			
PC 40:1; PC 18:1/22:0	2.54	.02			
PC 41:6; PC 19:0/22:6	5.16	.02			
PC 42:5	3.10	.02			
PE 37:7p; PE 16:0p/22:6	3.55	.02			
PE 38:6; PE 16:0/22:6; 16:1/22:5; 20:2/18:4	3.25	.04			
PE 40:6; PE 18:0/22:6	4.21	.05			
PS3 4:2	2.70	0.02			
Declined > twofold					
PA 10:0/18:3	0.41	.01	lysoPS 20:0	0.36	.04
PA 15:0/20:2	0.45	.02	PA 18:2/20:4	0.29	.05
PA 16:0/18:4	0.21	.00	PG 37:1	0.34	.01
PA 16:1/18:4	0.24	.00	PG 39:3	0.43	.01
PA 17:1/19:0	0.50	.02			
PA 18:3/21:0	0.46	.03			
PG 18:0/19:0	0.41	.01			
PS 38:1	0.47	.01			

## 4 DISCUSSION

The present study preliminarily found the differences of lipidomic profiles among patients of different lung cancer subtypes, genders, ages, stages, metastatic status, body qualities, and clinical phenome severities. Besides, it initially demonstrated clinical phenome‐associated lipid elements and lipid element‐associated clinical phenomes using clinical trans‐omics. Studies on lipidomic profiles of lung cancer patients have experienced three phases to detect the difference of lipidomic profiles between healthy and lung cancer patients,^16^ the association of multi‐omics among lung cancer subtypes,^3^ and the molecular mechanism of clinical lipidomics‐based target lipid elements.^17^ Of those lipidomics‐based data, limited information could be adopted to understand the disease occurrence and development, phone progression, and response to therapy, due to the lack of link between omics data and clinical phenomes. Like other omics investigations, most genomic data were not tied with clinical information, so that with little values to be understood and applied for clinical precision medicine.^18^ In order to face the major challenge that most clinical information was descriptive and unmatched with the digital quantity of omics data, clinical phenomes were scored by DESS and integrated with genomic and proteomic data of patients with acute respiratory distress syndrome and chronic obstructive pulmonary disease.^19^, ^20^, ^21^, ^22^ Clinical phenomes were furthermore integrated with lipidomic profiles in patients with pulmonary embolism, acute pneumonia, and acute exacerbation of chronic obstructive pulmonary diseases, based on clinical trans‐omics principle.^15^

Lipidomic profiles difference between health and lung cancer has been defined and it depends upon methodologies of measurement and analysis, sample preparations and sources, and patient populations and status.^8^ For example, serum levels of lysoPC (C26:0 and C26:1) and PC (C42:4 and C34:4) were different between stage I NSCLC and healthy patients.^22^ Some elements and pathways of serum PC and PE profiles increased in patients with lung benign disease and early‐stage NSCLC, as compared with healthy, whereas few (e.g., PC 15:0/18:1) significantly elevated in early‐stage NSCLC patients alone.^2^ It seems that patterns of lipid elements may be associated with the specificity of lung cancer and stage, rather than the intact lipid pathways. We performed a pilot prospective study to compare the variation of lipidomic profiles between health and lung cancer, and among lung cancer subtypes, as well as to correlate lipidomics with circulating leukocyte transcriptional profiles and clinical phenomes.^3^ The present study further investigated the difference of lipidomics among lung cancer subtypes with a special focus on subtype‐specific lipid elements. Of measured lipid elements, levels of PE elements (36:2, 18:0/18:2, and 18:1/18:1) were significantly higher in SCLC than in healthy control and other lung cancer subtypes, whereas lysoPC (20:1 and 22:0 sn‐position‐1) and PC (19:0/19:0 and 19:0/21:2) levels in ADC were more than 1500‐fold higher than in SCLC. Similar results were also seen in other studies: PCs (34:1, 36:2, 36:3, and 32:0) were found upregulated in NSCLC,^23^ whereas the latter (PC 32:0) was identified as a diagnostic factor for ADC in Hall's study.^24^ Marien also found an increase in several PC species.^25^

The specificity of human breast cancer cell subtypes was also noticed by measuring the quantitation of lipid C = C location and sn‐position structure isomers.^26^ The monounsaturated/saturated PC ratios were proposed to distinguish ADC histologic subtype‐dependent variations among lepidic, acinar, papillary, micropapillary, solid, and mucinous phenomes.^27^ The heterogeneity of lipidomic profiles and lipid metabolism could impact the specificity for lung cancer subtype, severity, stage, and drug efficacy, although characteristics of lipidomic profiles of lung cancer subtypes varied among studies because of different subjects, designs, methods, and analyses.^8^

In addition, multiple factors of lung cancer patients, for example, age, gender, stages, metastatic status, body condition, and clinical phenotyping, should be considered to evaluate values of lipidomic profiles. Lipidomic profiles with about 300 lipids in 11 classes in homogenized human lung cancer tissue were correlated with clinical and historical phenotypes, and it turned out that they had high links with stroma and tumor contents, inflammation, aging, and emphysema.^28^ Some of lung surfactant components changed in lung cancer tissues, including triacylglycerols, cholesteryl esters, and PGs. It was suggested that lipid panels might have the specificity to differentiate between ADC and SCC, and the association between lipid profiles of tumor‐free alveolar tissues and phenotypes. We found that the total number of changed lipid elements was obviously higher in younger patients, late stage, metastasis, or BMI ≤ 22. Of those changes, the decline number of lipids in male was 50% higher than female, <70 ages 60% than >70 ages, late stage 75% than early stage, metastasis 37% than nonmetastasis, or DESS > 90 58% than DESS ≤ 90.

Multidimensional analyses of lipidomic profiles in the present and previous studies strongly indicate that identified target panels of lipid elements have great impacts of identifying influencing factors in early diagnosis severity, progression, and therapeutic responses for lung cancer patients, by using multivariate classification or trans‐omic network models. Choline‐containing phospholipids of serum lipidomic profiles were considered as the potential biomarkers to differentiate between stage I NSCLC and noncancer subjects, especially lysoPC (C26:0 and C26:1) and PC (C42:4 and C34:4).^26^ The panel of lysoPC (18:0, 18:1, and 18:2) was suggested to detect early lung cancer patients from population screen, asymptomatic imaging detection, or stage IA‐IIB.^16^ We also selected several target panels with specificity on the basis of the fact that some lipids only altered in certain population of patients, for example, lysoPS (14:0, 16:0, 17:0, 18:3, and 20:1) and PS30:1 in male; PE (17:0/18:1, 17:0/18:2, 35:1, and 35:2) in old; PE (32:2 and 14:0/18:2) in early stage; PS35:5 in nonmetastasis; lysoPS18:2 and PS37:2 in high DESS; and lysoPC (14:0sn‐2, 16:1sn‐1, and 18:3sn‐1) and PE (38:6, 16:0/22:6, 16:1/22:5, 20:2/18:4, 40:6, 18:0/22:6, 40:7, 18:1/22:6, 42:8, and 20:2/22:6) in low BMI. Those panels need to be furthermore validated and defined in larger studies with more patients, clinical phenomes and lipidomic profiles, and well‐designed comparisons of related factors.

To differ from the correlation strategy between lipidomic profiles and other phenomes and reduce potential subjective biases, we furthermore analyzed the trans‐omic nodules cross lipidomic and clinical phenomic layer networks of mechanistic interactions, which is consisted of measures from patients with lung cancer subtypes with statistical difference from healthy patients. Yugi and Kuroda demonstrated that metabolism‐centric trans‐omics contained multiple nodules on each omic layer; cross multilayers presented global regulatory mechanisms of metabolic pathways, targets of direct/indirect regulations and interactions, and spatiotemporal dynamics of cell function‐dependent networks.^29^ Differentiated from multi‐omics, trans‐omics can be a potent approach to screen out and develop disease‐ and function‐specific biomarkers and therapeutic targets and provide more precise insights and predictions for disease progression and prognosis, although clinical applications are needed to evaluate the practical values, outcomes of trans‐nodules vary among methodologies, and repeatability and reliability of selected crossing nodules are validated.^4^, ^30^, ^31^ It is highly expected to link molecular omics with biological phenotypes, clinical phenomes, and environmental factors. We introduced clinical phenomics into trans‐omics to evaluate network nodules cross lipidomic and phenomic layers and define phenome‐specific lipid panels and lipid‐specific phenome groups in acute lung injury patients with or without infections and with or without chronic diseases, as well as in patients with lung cancer subtypes.^8^, ^15^, ^17^

To gather our present and previous studies, we found that the variation of trans‐omic profiles in patients was more obvious, uncontrolled, and irregular due to the complex and comprehensive influencing factors in clinical practice and measurements. We also found that the integrated panel appeared lung cancer subtype‐specific network and the construction to show statistical correlations and mechanistic molecular interactions between lipid metabolisms and phenome appearances in patients with lung cancer. For example, chest distress was corresponded with PS (33:2 and 37:5) and PG (35:4) in ADC and PS (40:1) in SCC, whereas sputum with PG 40:8 and lysoPS (22:6, 18:2, and 16:0) in ADC; PS(32:0 and 37:4) in SCC; and PG (40:6), lysoPS (15:0), and PS(30:0 and 30:1) in SCLC. It indicates that the same phenome in different diseases or disease subtypes may be generated from various metabolic pathways and metabolites. Oppositely, the same metabolic pathway and metabolites can contribute to the formation of different phenomes among diseases and disease subtypes. For example, lysoPS (16:0 and 22:6) were involved in the appearance of sputum, dysphagia, and hemoptysis in patients with ADC. The trans‐omic modules can reflect regulatory mechanisms by which cell survival and differentiation, organ function and metabolism, and systemic response and dysfunction are controlled.^17^, ^32^, ^33^ Those lipid metabolites and related limiting enzymes can be regulated by up/down‐expression of the corresponding genes and mitochondrial DNA methylation and fusion/fission.^34^, ^35^

There were also limitations in this study. First, the sample size was small, especially as for healthy control and for SCC and SCLC lung cancer groups. Second, therapeutic factors were not explored, because most lung cancer patients included underwent chemotherapy. For more and more effective molecular target medications and immunotherapies that were applied in lung cancer, corresponding groups of patients should be studied in future researches to explore medical influence on lipidomics. Last but not least, there might be latent confounding effects of complications to lipidomic metabolism, such as diabetes, hyperlipemia, and atherosclerosis, which were not strictly excluded in the study.

In conclusion, we qualitatively and quantitatively measured plasma lipidomic profiles in lung cancer patients and found that altered lipid panels and concentrations varied among lung cancer subtypes, genders, ages, stages, metastatic status, nutritional condition, and clinical phenome severity. Levels of PE elements (36:2, 18:0/18:2, and 18:1/18:1) were found to be SCLC specific and lysoPC (20:1 and 22:0 sn‐position‐1) and PC (19:0/19:0 and PC 19:0/21:2) ADC specific. We found that the declined number of lipids was obviously higher in male, <70 ages, late stage, metastasis, or DESS > 90. Clinical trans‐omics analyses demonstrated that one phenome in lung cancer subtypes may be generated from multiple metabolic pathways and metabolites, whereas a metabolic pathway and metabolite can contribute to different phenomes among subtypes, although those need to be furthermore confirmed by studies of larger population of patients in multicenters. Thus, our data suggested that trans‐omic profiles between clinical phenomes and lipidomes might have the value to uncover the heterogeneity of lipid metabolism among lung cancer subtypes and to find phenome‐based, subtype‐specific lipidomic biomarkers.

## Supporting information

Supporting information

## Data Availability

The data that support the findings of this study are available from the corresponding author upon reasonable request.
