# Supplementary material for: Trans‐omic profiling between clinical phenoms and lipidomes among patients with different subtypes of lung cancer
Source: Clin Transl Med. 2020 Aug 20;10(4):e151. doi: 10.1002/ctm2.151 (PMC7438979; doi:10.1002/ctm2.151)
Supplement: Supplementary file 1 — Supporting information [file CTM2-10-e151-s001.pdf]

Supplemental Table 1. Clinical phenomes scored and collected in each of three lung cancers, including patient histories, symptoms, signs, chemical measurements, image features, and pathologic indexes.

**Clinical phenomes**	**DESS Scores**			
	**0**	**1**	**2**	**4**
Symptoms				
Cough	no	slight	medium	severe
Sputum	no	slight	medium	severe
Hemoptysis	no	slight	medium	severe
Wheezing	no	slight	medium	severe
Dyspnea	no	slight	medium	severe
Dysphagia	no	slight	medium	severe
Chest distress	no	slight	medium	severe
Hoarse sound	no	slight	medium	severe
Chest pain	no	slight	medium	severe
Superior vena cava syn	no	slight	medium	severe
Horner syn	no	slight	medium	severe
Brachial plexus compression	no	slight	medium	severe
Fatigue	no	slight	medium	severe
Poor appetite	no	slight	medium	severe
Sleep	no	slight	medium	severe
Anxiety	no	slight	medium	severe
Urination defecation difficulties	no	slight	medium	severe
Weight loss	no	0-5%	5%-10%	>10%
PS score	0-1	2	3	4
Increased intracranial pressure	no	slight	medium	severe
Pain (bone)	no	slight	medium	severe
Pain (liver area)	no	slight	medium	severe
Osteoarthropathy hypertrophic pneumic	no	slight	medium	severe
Gynecomastia	no	slight	medium	severe
Cushing's syn	no	slight	medium	severe
SIADH	no	slight	medium	severe
Neuromuscular syn	no	slight	medium	severe
Carcinoid syn	no	slight	medium	severe
Multiple peripheral neuritis	no	slight	medium	severe
Myasthenia syn	no	slight	medium	severe
Signs				
Mind	normal	slight coma	medium coma	severe coma
Fever	normal	37.3-38.0℃	>38.0-39.0℃	>39℃
HR(bpm)	60-100			<60 or >100
RR	16-20			>20
BP(mmHg)	<140/<90	140-159/90-99	160-179/100-109	≥180/≥110
Jaundice	no	slight	medium	severe
Anemia	no	slight	medium	severe
Superficial LN	no	one	several	multiple location
Chest palpation	normal			abnormal
Chest percussion	normal			abnormal
Chest auscultation	normal			abnormal
Trachea in the middle	yes			no
Clear sound of both lungs	yes			no
Symmetrical respiratory sounds	yes			no
Velcro	no			yes

For Review Only

Crackle	no			yes
Heart	normal			abnormal sign
Abdomen	normal			abnormal sign
History				
Lung cancer family history	no			yes
Asthma	no	0-5 years	6-10 years	>10 years
COPD	no	0-5 years	6-10 years	>10 years
TB	no	0-5 years	6-10 years	>10 years
Other lung diseases	no	0-5 years	6-10 years	>10 years
Smoking (packet*year)	0	0-20	21-40	>40
Cancerogenic occupational environment	no	0-5 years	6-10 years	>10 years
HT	no	0-5 years	6-10 years	>10 years
DM	no	0-5 years	6-10 years	>10 years
CAD	no	0-5 years	6-10 years	>10 years
Other chronic disease	no	0-5 years	6-10 years	>10 years
Tumor lesion				
Pathological type	ADC	SCC	SCLC	Other
Differentiational degree	high	medium	low	undifferentiated
Stage (at Dx)	Ia & Ib	IIa-IIb	IIIa	IIIb & IV
Stage (now)	Ia & Ib	IIa-IIb	IIIa	IIIb & IV
T(Tumor)	Tx & T1	T2	T3	T4
Location	left			right
Left	upper lobe			down lobe
Right	upper lobe	middle lobe		down lobe
Sum of all T (mm)	0-30	31-50	51-70	>70
New lesion(T)	no	in same lobe		in other lobe
N (LN)	no	N1	N2	N3
N1(Ipsilateral paratracheal)	no			yes
N1(Ipsilateral hilum)	no			yes
N2(Below carina)	no			yes
N2(Ipsilateral mediastinum)	no			yes
N3(Supraclavicular)	no			yes
N3(Scalene muscle)	no			yes
N3(Opposite side)	no			yes
Sum of metastatic LN (mm)	0	1-20	>20-40	>40
New lesion(N)	no			yes
M (Pleural effusion)	no	slight	medium	severe
M (malignant pericardial effusion)	no	slight	medium	severe
M (Contralateral lung)	no	one	two	multiple
M (Brain)	no	one	two	multiple
M (Bone)	no	one	two	multiple
M (Liver)	no	one	two	multiple
M (Adrenal gland)	no	one	two	multiple
M (Other)	no	one	two	multiple
New lesion(metastasis)	no			yes
New metastasis location	bone	liver	brain	other
Therapy				
Neoadjuvant chemotherapy	no	3rd grade chemo		other
Neoadj chemo cycle	0	1	2	>2
Adjuvant chemotherapy	no	3rd grade chemo		other
Adju chemo cycle	0	1	2	>2
Postoperative recurrence time	none	>2 years	in two years	in one year
Treatment after recurrence	target therapy	chemotherapy	radiate therapy	other
Time to progress (TTP)	>24 months	in 12-24 months	in 6-12 months	in 6 months

For Review Only

First-line regimen	target therapy	chemotherapy	radiate therapy	other
First-line chemo regimen	3rd grade chemo	mono-chemo		other
First-line cycle	0	1--2	3--4	>4
Curative effect	CR	PR	SD	PD
Maintenance treatment	yes			no
Maintenance treatment regimen	target therapy	pemetrexed		other
First-line TTP	>24 months	12-24 months	6-12 months	in 6 months
Second-line regimen	target therapy	chemotherapy	radiate therapy	other
Second-line chemo regimen	pemetrexed	docetaxel		other
Second-line cycle	0	1--2	3--4	4--6
Second-line TTP	>9 months	7-9 months	4-6 months	in 3 months
Third-line regimen	chemotherapy	radiate therapy		other
Chemical measurements				
Hb(g/L)	Male: 160-120<br>Female: 150-110	Male: 90-119<br>Female: 90-109	60-89	≤59
WBC(×10^9/L)	≥4.0x10^9/L	3.9–3.0x10^9/L	2.9–2.0x10^9/L	<2.0x10^9/L
N(×10^9/L)	≥ 2.0x10^9/L	1.9–1.5x10^9 /L	1.4–1.0x10^9/L	<1.0x10^9 /L
PLT(×10^9/L)	≥100x10^9/L	99–75.0x10^9 /L	74.0–50.0x10^9 /L	<50.0x10^9 /L
Alb(g/L)	≥35	34-30	29-25	<25
ALT	≤ULN	>ULN–2.5xULN	>2.5–5.0xULN	>5.0xULN
AST	≤ULN	>ULN–2.5xULN	>2.5–5.0xULN	>5.0xULN
TB	≤ULN	>ULN–1.5xULN	>1.5–3.0xULN	>3.0xULN
CB	≤ULN	>ULN–1.5xULN	>1.5–3.0xULN	>3.0xULN
BUN (mmol/L)	3.2-7.1	7.2-9.0	9.1-19.9	>20
Cr (umol/L)	40-115	116-178	179-445	>445
UA (umol/L)	normal			>488(Male)<br>>387(Female)
Na(mmol/L)	136-145	146–150 or<br>135–130	151-155 or<br>131-125	>155 or <125
K(mmol/L)	3.5-5.2	3.0-3.4 or 5.3-5.5	2.5-2.9 or 5.6-6.0	<2.5 or >6.0
Cl (mmol/L)	96-105			>105 or <96
Ca(mmol/L)	2.15-2.55	>2.55-2.90 or<br><2.15-2.0	>2.9-3.1 or<br><2.0-1.75	>3.1-3.4 or<br><1.75-1.5
P (mmol/L)	0.9-1.34			>1.34 or <0.9
PH	>7.35	7.35-7.30	7.25-7.30	<7.25
PaO2(mmHg)	≥90	70-89	60-69	<60
PaCO2(mmHg)	35-44	45-47	48-50	>50
SCC	normal	>1xULN	>2xULN	>3xULN
CEA	normal	>1xULN	>2xULN	>3xULN
Cyfra211	normal	>1xULN	>2xULN	>3xULN
NSE	normal	>1xULN	>2xULN	>3xULN
CRP	normal	>1xULN	>2xULN	>3xULN
PT(s)	10.0-13.0	13.1-16		>16
FBG(mmol/L)	3.9-6.9	7.0-8.9 or 3.0-3.8	9.0-13.9 or 2.5-2.9	>14.0 or 2.0-2.4
Image				
Vacuole cavity	no			yes
Lobular	no			yes
Burr	no			yes
Obscure boundary	no			yes
Uneven density	no			yes
Pleural pull	no			yes
Pleural thickening	no			yes
Pleural effusion	no			yes

For Review Only

Enhancement	no			yes
Vascular involvement	no			yes
Bronchial stenosis	no			yes
Air bronchogram	no			yes
Obstructive pneumonia atelectasis	no			yes
Lymphangitis	no			yes
Pulmonary nodule	no			yes
Emphysema	no			yes
Bullae	no			yes
Bronchiectasis	no			yes
Patch exudation shadow	no			yes
Calcification	no			yes
Other image manifestation	no			yes
Pathology				
Surgical margin positive	no			yes
Pleural involvement	no			yes
Vascular involvement	no			yes
LN	no			yes
CK5/6	no			yes
CK7	no			yes
EGFR-E746	no			yes
EGFR-L858	no			yes
Ki-67	no			yes
NapsinA	no			yes
P40	no			yes
P63	no			yes
PD-1 tumor	no			yes
PD-1 interstitial	no			yes
PD-L1 tumor	no			yes
PD-L1 interstitial	no			yes
TTF-1	no			yes
Syn	no			yes
CD56	no			yes
CHG	no			yes
SPA	no			yes
LCA	no			yes
CEA	no			yes
NSE	no			yes
c-MET	no			yes
Her-2	no			yes
ALK Ventana	no			yes
ALK	no			yes
ROS-1	no			yes
RET	no			yes
EGFR	no			yes
K-ras	no			yes
N-ras	no			yes
B-raf	no			yes
PIK3CA	no			yes
HER-2	no			yes
Other indexes	no			yes
Complex	no			yes

For Review Only

Supplemental Table 2. Significantly elevated lipid elements of patients with adenocarcinoma, squamous cell carcinoma, or small cell lung cancer (more than two folds), as compared with healthy control (p values).

Adenocarcinoma			Squamous cell carcinoma			Small cell lung cancer		
**Lipids**	**Folds**	**P values**	**Lipids**	**Fold**	**P values**	**Lipids**	**Fold**	**P values**
lysoPC 16:0 (sn-2)	11.81	0.01	C1P240 Mean	7.46	0.05	lysoPG14:0	6.02	0.00
lysoPC 17:0 (sn-1)	11.33	0.01	d18:1S1P	6.11	0.04	lysoPG15:0	3.72	0.03
lysoPC 17:1 (sn-1)	6.92	0.02	d181So	11.88	0.05	lysoPI 15:1 (sn-1)	2.65	0.02
lysoPC 18:0 (sn-1)	6.45	0.02	lysoPC 19:0 (sn-1)	2.45	0.04	lysoPI 16:0 (sn-2)	5.38	0.02
lysoPC 18:1 (sn-1)	6.40	0.00	lysoPG15:0	4.71	0.04	lysoPI 17:0 (sn-1)	4.47	0.00
lysoPC 19:0 (sn-1)	5.99	0.05	lysoPI 20:3 (sn-1)	3.17	0.02	lysoPI 18:0 (sn-1)	6.40	0.02
lysoPC 20:0 (sn-1)	5.82	0.00	lysoPI 22:0 (sn-1)	7.51	0.04	lysoPI 18:1 (sn-1)	5.47	0.01
lysoPC 20:1 (sn-1)	5.79	0.00	lysoPI 22:4 (sn-1)	4.44	0.02	lysoPI 18:2 (sn-1)	5.41	0.04
lysoPC 20:4 (sn-1)	5.50	0.02	lysoPI 22:6 (sn-1)	3.97	0.00	lysoPI 18:3 (sn-1)	3.51	0.03
lysoPC 22:0 (sn-1)	5.21	0.00	lysoPS14:0	4.04	0.05	lysoPI 19:0 (sn-1)	7.29	0.00
lysoPC 22:6 (sn-1)	5.16	0.00	lysoPS17:0	3.38	0.04	lysoPI 20:0 (sn-1)	5.76	0.00
lysoPE19:0	5.14	0.03	lysoPS17:1	14.25	0.05	lysoPI 20:2 (sn-1)	7.60	0.00
lysoPG15:0	5.10	0.00	lysoPS18:3	5.25	0.02	lysoPI 20:3 (sn-1)	6.47	0.01
lysoPG16:0	4.66	0.01	PC 18:1/23:1	9.56	0.02	lysoPI 20:4 (sn-1)	3.91	0.01
lysoPG16:1	4.49	0.02	PC 39:3 (18:0/21:3)	2.46	0.00	lysoPI 22:0 (sn-1)	12.16	0.01
lysoPI 22:0 (sn-1)	4.42	0.03	PC 39:3; PC 19:0/20:3	2.56	0.00	lysoPI 22:4 (sn-1)	7.41	0.00
lysoPS14:0	4.40	0.03	PC 39:4 (18:0/21:4)	2.31	0.01	lysoPI 22:6 (sn-1)	9.70	0.00
lysoPS15:1	4.28	0.02	PC 39:5 (18:0/21:5)	2.11	0.01	lysoPS14:0	6.10	0.00
lysoPS16:0	4.26	0.01	PC 39:6; PC 17:0/22:6	2.12	0.00	lysoPS15:0	3.07	0.00
lysoPS18:2	4.23	0.01	PC 40:4; PC 18:0/22:4 or 20:1/20:3	2.10	0.01	lysoPS15:1	6.08	0.03
lysoPS18:3	4.06	0.00	PC 41:6; PC 19:0/22:6	2.96	0.00	lysoPS16:1	16.31	0.02
lysoPS20:0	3.98	0.02	PC 42:5	2.44	0.01	lysoPS17:0	4.43	0.01
lysoPS22:0	3.96	0.02	PE 35:5p; PE 16:0p/20:4	2.22	0.01	lysoPS17:1	17.04	0.01
lysoPS22:6	3.94	0.04	PE 35:6p; PE 16:0p/20:5	2.10	0.03	lysoPS18:1	3.37	0.02
PA 10:0/18:1	3.93	0.00	PE 36:1; PE 16:0/20:1 or 18:0/18:1	2.31	0.01	lysoPS18:3	6.92	0.01
PA 10:0/18:2	3.93	0.00	PE 36:5; PE 16:0/20:5	2.73	0.02	lysoPS19:0	4.37	0.01
PA 14:1/20:5	3.92	0.01	PE 37:6p; PE 18:0p/20:5 or 18:1p/20:4 or 16:0e/22:6	2.18	0.03	lysoPS20:1	3.05	0.01
PA 14:1/21:4	3.83	0.00	PE 37:7p; PE 16:0p/22:6	2.54	0.01	lysoPS20:2	4.73	0.02

For Review Only

PA 15:0/18:0	3.83	0.03	PE 38:1; PE 18:0/20:1	3.40	0.00	lysoPS20:4	3.22	0.05
PA 15:0/18:2	3.82	0.02	PE 38:2; PE 18:1/20:1	3.29	0.01	lysoPS20:5	3.80	0.01
PA 15:0/20:5	3.77	0.03	PE 38:3; PE 18:0/20:3	2.89	0.01	lysoPS22:0	3.47	0.03
PA 15:0/25:0	3.76	0.04	PE 38:5; PE 18:0/20:5	2.36	0.02	lysoPS22:4	4.85	0.02
PA 15:1/24:0	3.76	0.02	PE 38:7; PE 16:1/22:6 or 18:2/20:5	2.83	0.01	lysoPS22:6	3.67	0.03
PA 16:0/18:3	3.75	0.01	PE 39:7; PE 17:1/22:6	2.91	0.00	PA 14:0/20:5	20.60	0.03
PA 16:0/20:1	3.73	0.04	PE 40:1; PE 22:0/18:1	2.56	0.01	PA 14:0/24:5	26.32	0.04
PA 16:0/22:4	3.66	0.01	PE 40:2; PE 18:1/22:1	3.57	0.00	PC 18:1/23:1	3.34	0.00
PA 16:1/18:3	3.65	0.00	PE 40:3; PE 18:1/22:2 or 22:1/18:2	3.85	0.00	PC 41:6; PC 19:0/22:6	2.07	0.02
PA 16:1/18:4	3.63	0.00	PE 40:4; PE 18:0/22:4 or 20:0/20:4	2.52	0.02	PE 36:1; PE 16:0/20:1 or 18:0/18:1	3.28	0.03
PA 17:0/13:0	3.61	0.00	PE 40:6; PE 18:0/22:6	2.03	0.04	PE 36:2; PE 18:0/18:2 or 18:1/18:1	3.00	0.04
PA 17:0/20:5	3.53	0.03	PE 40:7; PE 18:1/22:6	2.91	0.01	PE 36:4; PE 16:0/20:4	3.32	0.04
PA 17:2/18:3	3.52	0.00	PE 42:8; PE 20:2/22:6	2.67	0.02	PE 37:6p; PE 18:0p/20:5 or 18:1p/20:4 or 16:0e/22:6	3.91	0.04
PA 18:0/20:1	3.51	0.01	PG30:0	4.09	0.01	PE 38:1; PE 18:0/20:1	5.27	0.01
PA 18:0/22:4	3.48	0.03	PG30:1	5.06	0.01	PE 38:2; PE 18:1/20:1	4.88	0.03
PA 18:1/18:3	3.46	0.00	PG31:0	3.68	0.00	PE 38:3; PE 18:0/20:3	3.86	0.03
PA 18:1/20:4	3.45	0.02	PG31:1	4.89	0.00	PE 38:4; PE 16:0/22:4	3.94	0.04
PA 18:2/20:4	3.45	0.04	PG32:0	3.16	0.01	PE 38:6; PE 16:0/22:6 or 16:1/22:5 or 20:2/18:4	4.60	0.02
PA 18:3/18:4	3.44	0.03	PG32:1	2.62	0.02	PE 39:7; PE 17:1/22:6	4.98	0.02
PA 18:3/20:4	3.34	0.03	PG32:2	2.89	0.01	PE 40:2; PE 18:1/22:1	5.00	0.02
PA 18:3/22:4	3.33	0.03	PG33:1	2.30	0.02	PE 40:3; PE 18:1/22:2 or 22:1/18:2	5.42	0.01
PA 18:4/19:0	3.30	0.02	PG34:0	3.41	0.01	PE 40:4; PE 18:0/22:4 or 20:0/20:4	4.01	0.02
PA 18:4/19:1	3.25	0.00	PG34:1	3.27	0.02	PG30:0	3.09	0.02
PA 18:4/20:5	3.24	0.03	PG34:4	2.41	0.01	PG30:1	6.81	0.00

For Review Only

PA 19:0/22:5	3.21	0.02	PG34:5	2.55	0.01	PG31:0	3.35	0.01
PA 19:0/23:0	3.16	0.02	PI 36:3; PI 16:0/20:3 or 18:0/18:3 or 18:1/18:2	8.10	0.00	PG31:1	3.72	0.00
PA 20:0/21:5	3.16	0.00	PS30:1	12.45	0.00	PG32:0	2.88	0.01
PA 20:4/26:2	3.11	0.00	PS31:0	4.60	0.01	PG32:1	2.72	0.02
PC 19:0/19:0	3.08	0.01	PS32:0	2.50	0.04	PG32:2	3.67	0.00
PC 19:0/21:2	3.05	0.02	PS34:3	2.74	0.00	PG33:0	2.58	0.01
PC 33:2e; PC 16:1e/18:1	3.02	0.02	PS34:4	3.29	0.00	PG33:1	2.34	0.02
PC 35:1; PC 16:0/19:1 or 17:0/18:1 or 17:1/18:0	3.02	0.00	PS34:5	2.91	0.00	PG34:0	3.20	0.01
PC 35:2e; PC 16:0e/20:2	2.90	0.00	PS35:1	2.36	0.00	PG34:1	3.51	0.01
PC 35:3; PC 17:1/18:2	2.88	0.02	PS35:2	2.23	0.04	PG34:4	2.59	0.01
PC 35:5; PC 15:0/20:5	2.83	0.04	PS36:3	2.39	0.04	PG34:5	3.47	0.01
PC 36:0p; PC 16:0p/22:6	2.81	0.01	PS36:5	2.93	0.03	PI 36:3; PI 16:0/20:3 or 18:0/18:3 or 18:1/18:2	12.09	0.01
PC 36:1; PC 18:0/18:1	2.77	0.00	PS37:3	3.11	0.01	PI 39:7; PI 17:1/22:6	2.05	0.04
PC 36:5; PC 14:0/22:5 or 16:0/20:5 or 16:1/20:4	2.74	0.02	PS37:6	3.95	0.00	PI 40:1; PI 18:1/22:0	15.74	0.01
PC 37:2; PC 18:1/19:1 or 18:2/19:0	2.74	0.02	PS38:7	2.97	0.03	PI 41:6; PI 19:0/22:6	2.76	0.03
PC 37:3; PC 17:0/20:3 or 19:1/18:2	2.72	0.00	TAG 53:2	3.46	0.01	PIP36:1	3.15	0.01
PC 37:4; PC 17:0/20:4	2.66	0.00	TAG 53:3	2.82	0.02	PS30:0	8.21	0.00
PC 37:5e; PC 16:0e/22:5 or 18:0e/20:5	2.66	0.00				PS30:1	13.36	0.00
PC 37:6; PC 15:0/22:6	2.66	0.02				PS31:0	8.23	0.00
PC 38:1; PC 18:1/20:0	2.54	0.01				PS31:1	5.10	0.01
PC 38:2; PC 16:0/22:2	2.51	0.01				PS32:1	5.53	0.01
PC 38:3; PC 18:0/20:3 or 18:1/20:2	2.49	0.01				PS33:2	2.11	0.03
PC 38:4; PC 18:0/20:4	2.47	0.02				PS34:2	3.10	0.01
PC 38:5; PC 18:0/20:5	2.45	0.02				PS34:3	2.55	0.00
PC 38:6; PC 16:0/22:6 or 18:2/20:4	2.45	0.01				PS34:4	2.35	0.02
PC 38:7; PC 16:1/22:6 or 18:2/20:5	2.41	0.02				PS34:5	2.51	0.01
PC 39:0 (18:0/21:0)	2.40	0.02				PS35:3	2.40	0.01
PC 39:2 (18:0/21:2)	2.38	0.03				PS36:6	2.55	0.01
PC 39:3 (18:0/21:3)	2.37	0.01				PS37:2	2.47	0.02
PC 39:3; PC 19:0/20:3	2.37	0.00				PS37:3	2.62	0.01
PC 39:4 (18:0/21:4)	2.34	0.01				PS38:7	2.22	0.02
PC 39:5 (18:0/21:5)	2.34	0.02				TAG 53:2	2.99	0.01
PC 39:6; PC 17:0/22:6	2.30	0.05				TAG 53:3	2.23	0.03
PC 39:7; PC 17:1/22:6	2.28	0.00				TAG 56:5	3.56	0.01

For Review Only

PC 40:1; PC 18:1/22:0	2.27	0.04							
PC 40:4; PC 18:0/22:4 or 20:1/20:3	2.26	0.02							
PC 40:5; PC 18:0/22:5	2.24	0.01							
PC 40:6; PC 18:0/22:6	2.24	0.02							
PC 40:7; PC 18:1/22:6	2.23	0.05							
PC 40:8; PC 20:4/20:4	2.23	0.00							
PC 41:6; PC 19:0/22:6	2.21	0.04							
PC 42:5	2.19	0.02							
PE 35:5p; PE 16:0p/20:4	2.17	0.03							
PE 35:6p; PE 16:0p/20:5	2.15	0.00							
PE 36:1; PE 16:0/20:1 or 18:0/18:1	2.13	0.03							
PE 36:5; PE 16:0/20:5	2.09	0.00							
PE 36:6; PE 16:1/20:5	2.06	0.02							
PE 37:6p; PE 18:0p/20:5 or 18:1p/20:4 or 16:0e/22:6	2.06	0.00							
PE 37:7p; PE 16:0p/22:6	2.04	0.04							
PE 38:1; PE 18:0/20:1	2.03	0.02							
PE 38:2; PE 18:1/20:1	2.03	0.00							

For Review Only

Supplemental Table 3. Significantly declined lipid elements of patients with adenocarcinoma, squamous cell carcinoma, or small cell lung cancer (more than two folds), as compared with healthy control (p values).

Adenocarcinoma			Squamous cell carcinoma			Small cell lung cancer		
**Lipids**	**Folds**	**P values**	**Lipids**	**Folds**	**P values**	**Lipids**	**Folds**	**P values**
PG36:6	0.50	0.01	PA 10:0/18:1	0.43	0.03	PA 10:0/18:2	0.30	0.01
PG37:1	0.50	0.00	PA 10:0/18:2	0.40	0.01	PA 10:0/18:3	0.31	0.04
PG37:6	0.49	0.05	PA 14:1/20:5	0.09	0.00	PA 14:1/20:5	0.17	0.01
PG39:3	0.48	0.03	PA 14:1/21:4	0.22	0.01	PA 14:1/21:4	0.17	0.02
PG40:6	0.48	0.00	PA 15:0/18:0	0.45	0.04	PA 15:0/20:2	0.29	0.04
PG40:8	0.47	0.00	PA 15:0/20:5	0.05	0.01	PA 15:0/20:5	0.10	0.03
PS30:0	0.46	0.02	PA 15:0/25:0	0.10	0.01	PA 15:0/25:0	0.08	0.02
PS30:1	0.44	0.01	PA 15:1/24:0	0.27	0.01	PA 15:1/24:0	0.31	0.04
PS31:0	0.44	0.00	PA 16:0/18:3	0.25	0.00	PA 16:0/18:3	0.25	0.01
PS31:1	0.43	0.00	PA 16:0/20:1	0.45	0.02	PA 16:0/18:4	0.17	0.03
PS32:0	0.40	0.00	PA 16:0/22:4	0.46	0.04	PA 16:0/22:4	0.34	0.03
PS33:0	0.39	0.01	PA 16:1/18:3	0.28	0.01	PA 16:1/18:3	0.21	0.02
PS33:2	0.37	0.04	PA 17:0/13:0	0.38	0.04	PA 16:1/18:4	0.19	0.03
PS34:2	0.37	0.00	PA 17:0/20:5	0.29	0.03	PA 18:0/18:4	0.39	0.02
PS34:3	0.37	0.01	PA 18:0/20:1	0.49	0.02	PA 18:0/20:1	0.38	0.01
PS34:4	0.37	0.02	PA 18:0/22:4	0.44	0.02	PA 18:0/22:4	0.34	0.01
PS34:5	0.34	0.00	PA 18:2/18:4	0.46	0.01	PA 18:1/ 18:4	0.49	0.02
PS35:1	0.30	0.00	PA 18:3/18:4	0.32	0.01	PA 18:1/18:4	0.47	0.02
PS35:2	0.29	0.00	PA 18:3/20:4	0.48	0.01	PA 18:1/22:4	0.46	0.02
PS35:3	0.27	0.00	PA 18:4/19:1	0.22	0.02	PA 18:2/18:4	0.50	0.03
PS36:4	0.25	0.01	PA 18:4/20:2	0.32	0.03	PA 18:3/18:4	0.23	0.01
PS36:5	0.24	0.04	PA 18:4/20:4	0.38	0.00	PA 18:3/20:0	0.37	0.02
PS36:6	0.23	0.00	PA 18:4/20:5	0.46	0.01	PA 18:3/20:4	0.37	0.01
PS37:3	0.22	0.00	PA 19:0/22:5	0.19	0.02	PA 18:3/22:4	0.26	0.01
PS37:5	0.20	0.00	PA 19:0/23:0	0.16	0.01	PA 18:4/19:1	0.11	0.02
PS37:6	0.19	0.00	PA 20:0/21:5	0.13	0.01	PA 18:4/20:1	0.43	0.02
PS38:7	0.15	0.00	PG 18:0/19:0	0.44	0.03	PA 18:4/20:2	0.21	0.03
PS41:6	0.11	0.00	PG37:1	0.34	0.04	PA 18:4/20:4	0.31	0.01
SM240	0.05	0.00	PG39:3	0.41	0.05	PA 19:0/22:5	0.17	0.03
			PG40:6	0.43	0.02	PA 19:0/23:0	0.13	0.03
			PG40:8	0.39	0.02	PA 20:0/21:5	0.17	0.02
			PS40:1	0.30	0.04	PA 20:4/26:2	0.37	0.01
						PG40:6	0.42	0.04
						PS41:6	0.39	0.01

For Review Only

Supplemental Table 4. Significantly elevated lipid elements of male and female patients with lung cancer (more than two folds), as compared with healthy control (p values)

Male patients with lung cancer			Female patients with lung cancer		
**Lipids**	**Folds**	**P values**	**Lipids**	**Folds**	**P values**
lysoPC 18:0 (sn-1)	2.72	0.04	C1P120 Mean	4.27	0.03
lysoPC 19:0 (sn-1)	2.75	0.01	C1P160 Mean	8.00	0.03
lysoPC 20:0 (sn-1)	2.38	0.04	C1P240 Mean	11.5	0.01
lysoPC 22:0 (sn-1)	2.81	0.04	Cer120	4.29	0.04
lysoPG15:0	4.13	0.03	d171So	4.19	0.04
lysoPG16:1	2.87	0.04	d18:0Sa1P	3.64	0.04
lysoPI 20:3 (sn-1)	4.82	0.03	d18:1S1P	4.82	0.05
lysoPI 22:0 (sn-1)	12.05	0.01	d181So	6.86	0.02
lysoPS14:0	5.03	0.01	lysoPC 18:0 (sn-1)	2.42	0.04
lysoPS16:0	5.07	0.01	lysoPC 19:0 (sn-1)	2.51	0.03
lysoPS17:0	3.64	0.01	lysoPC 20:0 (sn-1)	2.26	0.03
lysoPS17:1	12.47	0.04	lysoPC 22:0 (sn-1)	2.7	0.04
lysoPS18:1	3.77	0.03	lysoPC 22:6 (sn-1)	2.67	0.04
lysoPS18:2	5.63	0.02	lysoPG15:0	5.93	0.03
lysoPS18:3	6.24	0.01	lysoPG16:1	2.99	0.01
lysoPS20:1	2.69	0.02	lysoPI 18:0 (sn-1)	2.8	0.01
lysoPS20:2	3.80	0.03	lysoPI 18:1 (sn-1)	3.48	0.02
lysoPS20:5	3.00	0.04	lysoPI 20:2 (sn-1)	5.16	0.04
lysoPS22:0	3.19	0.05	lysoPI 22:0 (sn-1)	7.23	0.01
PC 37:3; PC 17:0/20:3 or 19:1/18:2	2.07	0.03	lysoPI 22:6 (sn-1)	7.00	0.00
PC 37:4; PC 17:0/20:4	2.09	0.01	lysoPS14:0	2.38	0.03
PC 37:5e; PC 16:0e/22:5 or 18:0e/20:5	2.03	0.01	lysoPS15:1	3.64	0.02
PC 39:2 (18:0/21:2)	2.62	0.04	lysoPS18:2	4.02	0.03
PC 39:3 (18:0/21:3)	3.44	0.03	lysoPS20:5	2.32	0.03
PC 39:3; PC 19:0/20:3	3.43	0.03	lysoPS22:0	2.29	0.03
PC 39:4 (18:0/21:4)	3.30	0.02	PC 16:0/26:0	2.42	0.03
PC 39:5 (18:0/21:5)	2.93	0.02	PC 18:1/23:1	6.85	0.01
PC 39:6; PC 17:0/22:6	3.09	0.01	PC 39:0 (18:0/21:0)	2.34	0.00
PC 39:7; PC 17:1/22:6	2.63	0.00	PC 39:7; PC 17:1/22:6	2.48	0.03
PC 40:1; PC 18:1/22:0	2.47	0.04	PC 40:4; PC 18:0/22:4 or 20:1/20:3	2.25	0.04
PC 40:4; PC 18:0/22:4 or 20:1/20:3	2.36	0.00	PC 40:5; PC 18:0/22:5	2.1	0.01
PC 40:5; PC 18:0/22:5	2.09	0.00	PC 40:6; PC 18:0/22:6	2.1	0.00
PC 41:6; PC 19:0/22:6	4.81	0.04	PC 40:7; PC 18:1/22:6	2.21	0.00
PC 42:5	2.89	0.01	PC 40:8; PC 20:4/20:4	2.03	0.01
PE 36:1; PE 16:0/20:1 or 18:0/18:1	2.51	0.02	PE 35:5p; PE 16:0p/20:4	2.24	0.02
PE 38:1; PE 18:0/20:1	3.68	0.01	PE 35:6p; PE 16:0p/20:5	2.49	0.02
PE 38:2; PE 18:1/20:1	3.55	0.02	PE 36:1; PE 16:0/20:1 or 18:0/18:1	2.43	0.02
PE 38:3; PE 18:0/20:3	3.08	0.01	PE 36:5; PE 16:0/20:5	2.08	0.04
PE 39:7; PE 17:1/22:6	3.51	0.02	PE 37:6p; PE 18:0p/20:5 18:1p/20:4 or 16:0e/22:6	2.34	0.02
PE 40:2; PE 18:1/22:1	4.32	0.00	PE 37:7p; PE 16:0p/22:6	2.56	0.01
PE 40:3; PE 18:1/22:2 or 22:1/18:2	4.54	0.00	PE 38:1; PE 18:0/20:1	3.68	0.01
PE 40:4; PE 18:0/22:4 r 20:0/20:4	2.95	0.02	PE 38:2; PE 18:1/20:1	3.37	0.02
PE 40:7; PE 18:1/22:6	3.81	0.03	PE 38:3; PE 18:0/20:3	2.99	0.02
PG30:0	4.97	0.00	PE 38:5; PE 18:0/20:5	2.22	0.04
PG30:1	6.67	0.00	PE 38:7; PE 16:1/22:6 ;18:2/20:5	2.56	0.03

For Review Only

PG31:0	4.82	0.00	PE 39:7; PE 17:1/22:6	3.25	0.01
PG31:1	5.22	0.00	PE 40:2; PE 18:1/22:1	4.01	0.02
PG32:0	3.53	0.00	PE 40:3; PE 18:1/22:2 or 22:1/18:2	4.02	0.02
PG32:1	2.91	0.00	PE 40:4; PE 18:0/22:4 or 20:0/20:4	2.9	0.03
PG32:2	3.18	0.00	PG30:0	4.36	0.01
PG33:0	2.59	0.00	PG30:1	4.99	0.00
PG33:1	2.47	0.00	PG31:0	3.58	0.00
PG34:0	3.87	0.00	PG31:1	3.83	0.00
PG34:1	3.96	0.00	PG32:0	3.31	0.00
PG34:2	2.02	0.01	PG32:1	2.94	0.00
PG34:4	3.16	0.00	PG32:2	2.44	0.00
PG34:5	3.51	0.00	PG33:0	2.47	0.01
PG36:1	2.38	0.01	PG33:1	2.54	0.00
PG36:2	2.27	0.02	PG34:0	3.16	0.00
PI 31:1p; PI 16:0p/16:0	2.39	0.04	PG34:1	3.25	0.00
PS30:0	8.78	0.02	PG34:2	2.36	0.00
PS30:1	14.81	0.00	PG34:4	2.92	0.03
PS31:0	6.29	0.00	PG34:5	3.34	0.03
PS31:1	6.20	0.01	PG36:6	2.54	0.01
PS32:0	3.41	0.02	PI 36:3;PI 16:0/20:3;18:0/18:3; 18:1/18:2	8.33	0.00
PS32:1	6.28	0.02	PS30:0	3.87	0.00
PS33:2	2.23	0.01	PS30:1	7.28	0.00
PS34:2	3.21	0.02	PS31:0	3.54	0.01
PS34:3	3.25	0.00	PS31:1	3.72	0.00
PS34:4	3.38	0.00	PS34:3	2.73	0.00
PS34:5	3.29	0.00	PS34:4	3.03	0.00
PS35:1	2.07	0.02	PS34:5	2.86	0.00
PS35:2	2.08	0.01	PS35:5	2.32	0.03
PS35:3	3.11	0.00	PS36:5	3.44	0.01
PS36:5	3.31	0.05	PS36:6	3.34	0.00
PS36:6	2.93	0.04	PS37:2	2.23	0.03
PS37:3	2.97	0.01	PS37:6	3.17	0.00
PS37:5	2.12	0.01	PS38:7	2.08	0.02
PS37:6	3.70	0.00			
PS38:7	3.62	0.02			

For Review Only

Supplemental Table 5. Significantly declined lipid elements of male and female patients with lung cancer (more than two folds), as compared with healthy control (p values)

Male patients with lung cancer			Female patients with lung cancer		
**Lipids**	**Folds**	**P values**	**Lipids**	**Folds**	**P values**
lysoPS20:0	0.45	0.04	PA 10:0/18:2	0.38	0.00
PA 10:0/18:1	0.40	0.00	PA 14:1/20:5	0.12	0.00
PA 10:0/18:2	0.40	0.00	PA 14:1/21:4	0.25	0.01
PA 14:1/20:5	0.11	0.00	PA 15:0/20:5	0.05	0.00
PA 14:1/21:4	0.25	0.00	PA 15:0/25:0	0.15	0.01
PA 15:0/18:0	0.49	0.01	PA 15:1/24:0	0.43	0.03
PA 15:0/20:2	0.47	0.01	PA 16:0/18:3	0.28	0.00
PA 15:0/20:5	0.07	0.00	PA 16:0/18:4	0.25	0.01
PA 15:0/25:0	0.11	0.00	PA 16:1/18:3	0.27	0.01
PA 15:1/24:0	0.26	0.00	PA 16:1/18:4	0.32	0.02
PA 16:0/18:3	0.25	0.00	PA 17:0/20:5	0.36	0.04
PA 16:0/20:1	0.46	0.00	PA 18:0/22:4	0.50	0.03
PA 16:0/22:4	0.44	0.00	PA 18:3/18:4	0.36	0.01
PA 16:1/18:3	0.28	0.00	PA 18:3/22:4	0.48	0.03
PA 17:0/13:0	0.37	0.00	PA 18:4/19:0	0.34	0.05
PA 17:0/20:5	0.31	0.00	PA 18:4/19:1	0.21	0.01
PA 18:0/20:1	0.47	0.00	PA 19:0/22:5	0.25	0.01
PA 18:0/22:4	0.43	0.00	PA 19:0/23:0	0.22	0.01
PA 18:1/20:4	0.25	0.00	PA 20:0/21:5	0.20	0.00
PA 18:2/20:4	0.26	0.02	PG40:6	0.44	0.01
PA 18:3/18:4	0.31	0.00	PG40:8	0.39	0.01
PA 18:3/20:4	0.47	0.00			
PA 18:3/22:4	0.40	0.00			
PA 18:4/19:0	0.35	0.01			
PA 18:4/19:1	0.20	0.00			
PA 18:4/20:2	0.39	0.01			
PA 18:4/20:4	0.48	0.00			
PA 19:0/22:5	0.18	0.00			
PA 19:0/23:0	0.15	0.00			
PA 20:0/21:5	0.15	0.00			
PA 20:4/26:2	0.40	0.00			
PG 18:0/19:0	0.42	0.00			
PG37:1	0.39	0.01			
PG40:6	0.46	0.00			
PG40:8	0.40	0.00			
PS38:1	0.46	0.00			
PS40:1	0.36	0.01			
PS41:6	0.45	0.00			

For Review Only

Supplemental Table 6. Significantly elevated lipid elements of patients with lung cancer at different ages (more than two folds), as compared with healthy control (p values)

Patient age < 60			Patient age 60-70			Patient age > 70		
**Lipids**	**Folds**	**P Values**	**Lipids**	**Folds**	**P Values**	**Lipids**	**Folds**	**P Values**
C1P120 Mean	20.07	0.04	lysoPG14:0	6.28	0.00	d18:1S1P	7.97	0.04
lysoPC 15:0 (sn-2)	3.11	0.03	lysoPG15:0	3.23	0.03	lysoPC 16:0 (sn-2)	3.25	0.05
lysoPC 15:1 (sn-1)	6.94	0.02	lysoPI 16:0 (sn-2)	3.89	0.04	lysoPC 17:1 (sn-1)	2.65	0.04
lysoPC 16:0 (sn-2)	3.78	0.04	lysoPI 17:0 (sn-1)	3.16	0.02	lysoPC 18:0 (sn-1)	3.09	0.02
lysoPC 16:1 (sn-1)	3.67	0.02	lysoPI 18:0 (sn-1)	4.99	0.04	lysoPC 18:1 (sn-1)	3.14	0.05
lysoPC 17:0 (sn-1)	3.53	0.01	lysoPI 18:1 (sn-1)	4.42	0.02	lysoPC 19:0 (sn-1)	3.49	0.00
lysoPC 17:1 (sn-1)	4.03	0.02	lysoPI 18:3 (sn-1)	2.90	0.04	lysoPC 20:0 (sn-1)	3.19	0.01
lysoPC 18:0 (sn-1)	3.42	0.01	lysoPI 19:0 (sn-1)	6.62	0.00	lysoPC 20:1 (sn-1)	2.99	0.01
lysoPC 18:1 (sn-1)	3.91	0.04	lysoPI 20:0 (sn-1)	4.36	0.01	lysoPC 20:3 (sn-1)	2.45	0.02
lysoPC 18:2 (sn-1)	5.61	0.05	lysoPI 20:2 (sn-1)	6.76	0.02	lysoPC 20:4 (sn-1)	3.12	0.02
lysoPC 18:3 (sn-1)	4.93	0.02	lysoPI 20:3 (sn-1)	5.34	0.01	lysoPC 20:5 (sn-1)	2.71	0.01
lysoPC 19:0 (sn-1)	3.26	0.00	lysoPI 20:4 (sn-1)	2.99	0.03	lysoPC 22:0 (sn-1)	3.77	0.01
lysoPC 20:0 (sn-1)	2.63	0.01	lysoPI 22:0 (sn-1)	10.88	0.00	lysoPC 22:4 (sn-1)	2.24	0.03
lysoPC 20:1 (sn-1)	2.35	0.01	lysoPI 22:4 (sn-1)	6.56	0.00	lysoPC 22:6 (sn-1)	3.14	0.01
lysoPC 20:2 (sn-1)	3.29	0.05	lysoPI 22:6 (sn-1)	6.91	0.00	lysoPE19:0	7.21	0.01
lysoPC 20:3 (sn-1)	3.80	0.04	lysoPS14:0	6.13	0.00	lysoPE19:1	3.3	0.05
lysoPC 20:4 (sn-1)	3.87	0.03	lysoPS15:0	2.92	0.01	lysoPG16:1	2.23	0.03
lysoPC 20:5 (sn-1)	6.50	0.03	lysoPS16:0	4.17	0.05	lysoPI 22:0 (sn-1)	10.5	0.05
lysoPC 22:0 (sn-1)	3.37	0.01	lysoPS16:1	9.51	0.01	lysoPS18:2	3.5	0.02
lysoPC 22:6 (sn-1)	4.12	0.02	lysoPS17:0	3.83	0.02	PC 16:0/26:0	3.82	0.00
lysoPG15:0	7.77	0.01	lysoPS17:1	14.14	0.02	PC 18:1/23:1	9.45	0.02
lysoPG15:1	2.74	0.04	lysoPS18:2	5.13	0.04	PC 19:0/19:0	2.99	0.00
lysoPG16:0	3.01	0.02	lysoPS18:3	6.80	0.00	PC 19:0/21:2	3.78	0.00
lysoPG16:1	4.50	0.00	lysoPS20:1	3.01	0.01	PC 37:3; PC 17:0/20:3 or 19:1/18:2	2.23	0.05
lysoPG18:3	3.08	0.02	lysoPS20:2	4.61	0.02	PC 37:4; PC 17:0/20:4	2.32	0.04
lysoPI 18:0 (sn-1)	3.76	0.04	lysoPS20:5	3.84	0.01	PC 37:5e; PC 16:0e/22:5 or 18:0e/20:5	2.17	0.03
lysoPI 20:2 (sn-1)	5.18	0.03	lysoPS22:0	3.77	0.01	PC 39:0 (18:0/21:0)	2.21	0.00
lysoPI 22:0 (sn-1)	9.91	0.02	lysoPS22:6	2.85	0.04	PC 39:3; PC 19:0/20:3	3.9	0.05
lysoPI 22:6 (sn-1)	8.30	0.01	PA 14:0/20:5	13.29	0.05	PC 39:6; PC 17:0/22:6	3.38	0.03
lysoPS14:0	3.74	0.05	PC 18:1/23:1	7.12	0.05	PC 39:7; PC 17:1/22:6	2.84	0.02
lysoPS15:1	5.27	0.00	PC 39:3 (18:0/21:3)	2.07	0.01	PC 40:1; PC 18:1/22:0	2.65	0.04
lysoPS16:0	3.93	0.01	PC 39:3; PC 19:0/20:3	2.02	0.02	PC 40:4; PC 18:0/22:4 or 20:1/20:3	2.61	0.02

For Review Only

lysoPS17:1	14.16	0.03	PC 39:4 (18:0/21:4)	2.16	0.00	PC 40:5; PC 18:0/22:5	2.36	0.00
lysoPS18:1	3.72	0.05	PC 39:6; PC 17:0/22:6	2.06	0.00	PC 40:6; PC 18:0/22:6	2.17	0.00
lysoPS18:2	6.47	0.02	PC 41:6; PC 19:0/22:6	2.50	0.00	PC 40:7; PC 18:1/22:6	2.11	0.00
lysoPS18:3	4.03	0.03	PE 35:5p; PE 16:0p/20:4	2.76	0.05	PC 40:8; PC 20:4/20:4	2.29	0.01
lysoPS19:0	5.66	0.04	PE 36:1; PE 16:0/20:1 or 18:0/18:1	3.02	0.01	PC 41:6; PC 19:0/22:6	5.27	0.03
lysoPS20:3	2.33	0.02	PE 36:2; PE 18:0/18:2 or 18:1/18:1	2.51	0.04	PC 42:5	3.65	0.04
lysoPS20:5	2.54	0.05	PE 36:4; PE 16:0/20:4	2.76	0.04	PE 35:5p; PE 16:0p/20:4	2.31	0.00
lysoPS22:6	4.30	0.05	PE 37:7p; PE 16:0p/22:6	3.43	0.05	PE 35:6p; PE 16:0p/20:5	2.22	0.02
PC 37:3; PC 17:0/20:3 or 19:1/18:2	2.33	0.03	PE 38:1; PE 18:0/20:1	4.18	0.02	PE 36:1; PE 16:0/20:1 or 18:0/18:1	2.36	0.02
PC 37:4; PC 17:0/20:4	2.34	0.02	PE 38:2; PE 18:1/20:1	4.14	0.02	PE 36:5; PE 16:0/20:5	2.2	0.04
PC 37:5e; PC 16:0e/22:5 or 18:0e/20:5	2.21	0.02	PE 38:3; PE 18:0/20:3	3.55	0.01	PE 37:3; PE 17:0/20:3	2.53	0.02
PC 39:0 (18:0/21:0)	2.16	0.00	PE 38:4; PE 16:0/22:4	3.26	0.03	PE 37:6p; PE 18:0p/20:5 or 18:1p/20:4;16:0e/22:6	2.31	0.02
PC 39:2 (18:0/21:2)	3.06	0.03	PE 38:6; PE 16:0/22:6 or 16:1/22:5;20:2/18:4	3.69	0.02	PE 37:7p; PE 16:0p/22:6	2.41	0.02
PC 39:3 (18:0/21:3)	4.28	0.02	PE 39:7; PE 17:1/22:6	4.16	0.01	PE 38:1; PE 18:0/20:1	3.73	0.01
PC 39:3; PC 19:0/20:3	4.31	0.02	PE 40:2; PE 18:1/22:1	4.45	0.01	PE 38:2; PE 18:1/20:1	3.45	0.02
PC 39:4 (18:0/21:4)	3.89	0.02	PE 40:3; PE 18:1/22:2 or 22:1/18:2	4.77	0.01	PE 38:3; PE 18:0/20:3	2.91	0.02
PC 39:5 (18:0/21:5)	3.44	0.02	PE 40:4; PE 18:0/22:4 or 20:0/20:4	3.43	0.01	PE 38:7; PE 16:1/22:6 or 18:2/20:5	2.79	0.02
PC 39:6; PC 17:0/22:6	3.70	0.01	PE 40:7; PE 18:1/22:6	4.13	0.03	PE 39:7; PE 17:1/22:6	3.21	0.02
PC 39:7; PC 17:1/22:6	3.02	0.01	PG30:0	5.10	0.01	PE 40:1; PE 22:0/18:1	4.22	0.03
PC 40:1; PC 18:1/22:0	2.97	0.03	PG30:1	5.88	0.00	PE 40:2; PE 18:1/22:1	4.37	0.02
PC 40:4; PC 18:0/22:4 or 20:1/20:3	2.45	0.00	PG31:0	4.34	0.00	PE 40:3; PE 18:1/22:2 or 22:1/18:2	4.34	0.01
PC 40:5; PC 18:0/22:5	2.07	0.00	PG31:1	4.77	0.00	PE 40:4; PE 18:0/22:4 or 20:0/20:4	2.94	0.03
PC 40:8; PC 20:4/20:4	2.09	0.02	PG32:0	3.47	0.00	PE 40:7; PE 18:1/22:6	3.29	0.03

For Review Only

PC 41:6; PC 19:0/22:6	6.31	0.03	PG32:1	2.81	0.00	PE 42:8; PE 20:2/22:6	3.13	0.03
PC 42:5	2.90	0.01	PG32:2	2.99	0.00	PG30:0	3.11	0.02
PE 36:5; PE 16:0/20:5	2.73	0.04	PG33:0	2.40	0.01	PG30:1	4.27	0.00
PE 38:1; PE 18:0/20:1	3.08	0.01	PG33:1	2.44	0.00	PG31:0	2.4	0.01
PE 38:2; PE 18:1/20:1	2.80	0.04	PG34:0	3.82	0.00	PG31:1	3.26	0.00
PE 38:3; PE 18:0/20:3	2.61	0.04	PG34:1	3.91	0.01	PG32:0	2.8	0.00
PE 38:5; PE 18:0/20:5	2.38	0.05	PG34:2	2.08	0.01	PG32:1	2.45	0.02
PE 39:7; PE 17:1/22:6	2.79	0.02	PG34:4	2.50	0.01	PG33:0	2.08	0.03
PE 40:1; PE 22:0/18:1	3.60	0.03	PG34:5	2.75	0.02	PG33:1	2.1	0.02
PE 40:2; PE 18:1/22:1	3.82	0.01	PG36:1	2.01	0.01	PG34:0	2.82	0.01
PE 40:3; PE 18:1/22:2 or 22:1/18:2	3.94	0.01	PI 31:1p; PI 16:0p/16:0	2.04	0.02	PG34:1	2.77	0.01
PG30:0	5.88	0.00	PI 36:3; PI 16:0/20:3 or 18:0/18:3 or 18:1/18:2	12.24	0.00	PG34:4	2.3	0.01
PG30:1	8.00	0.00	PI 40:1; PI 18:1/22:0	11.56	0.03	PG34:5	2.13	0.05
PG31:0	6.26	0.00	PI 41:6; PI 19:0/22:6	2.26	0.04	PG35:1	2.27	0.02
PG31:1	6.09	0.00	PIP36:1	2.40	0.04	PS30:0	4.11	0.01
PG32:0	4.02	0.00	PS30:0	9.29	0.00	PS30:1	7.12	0.00
PG32:1	3.45	0.00	PS30:1	17.23	0.00	PS31:0	2.94	0.00
PG32:2	3.69	0.00	PS31:0	7.58	0.00	PS31:1	4.98	0.00
PG33:0	3.13	0.00	PS31:1	5.80	0.01	PS34:3	3.02	0.00
PG33:1	2.90	0.00	PS32:0	2.69	0.03	PS34:4	3.07	0.00
PG33:2	2.11	0.01	PS32:1	5.30	0.01	PS34:5	2.9	0.00
PG34:0	4.15	0.00	PS32:2	4.17	0.05	PS35:4	2.62	0.04
PG34:1	4.37	0.00	PS33:2	2.14	0.04	PS36:5	3.15	0.01
PG34:2	2.43	0.01	PS34:2	3.29	0.00	PS36:6	2.46	0.03
PG34:4	4.41	0.00	PS34:3	2.67	0.00	PS37:2	2.64	0.02
PG34:5	5.41	0.00	PS34:4	2.65	0.00	PS37:6	3.37	0.01
PG35:2	2.28	0.03	PS34:5	2.53	0.00	PS38:7	3.65	0.04
PG35:4	2.33	0.04	PS35:2	2.07	0.02			
PG36:1	2.65	0.01	PS35:3	2.87	0.00			
PG36:2	2.55	0.01	PS36:6	3.53	0.04			
PG36:6	2.54	0.01	PS37:3	3.11	0.01			
PI 36:3; PI 16:0/20:3 or 18:0/18:3 or 18:1/18:2	8.33	0.00	PS37:6	3.51	0.00			
PS30:1	11.43	0.00	TAG 53:2	4.00	0.00			
PS31:0	5.09	0.01						
PS31:1	5.24	0.02						
PS32:0	4.32	0.01						
PS32:1	5.15	0.03						
PS32:2	2.93	0.04						
PS33:2	2.21	0.01						
PS34:3	3.59	0.00						
PS34:4	4.12	0.00						

For Review Only

PS34:5	4.05	0.00						
PS35:1	2.30	0.00						
PS35:2	2.24	0.00						
PS35:3	3.17	0.01						
PS36:5	3.27	0.02						
PS36:6	3.08	0.00						
PS37:3	2.54	0.05						
PS37:5	2.22	0.02						
PS37:6	3.68	0.00						
PS38:7	2.79	0.01						

For Review Only

Supplemental Table 7. Significantly declined lipid elements of patients with lung cancer at different ages (more than two folds), as compared with healthy control (p values)

Patient age < 60			Patient age 60-70			Patient age > 70		
**Lipids**	**Folds**	**P Values**	**Lipids**	**Folds**	**P Values**	**Lipids**	**Folds**	**P Values**
lysoPE 16:0 (sn-1)	0.41	0.04	PA 10:0/18:1	0.43	0.02	PA 10:0/18:2	0.49	0.02
lysoPE 18:0 (sn-1)	0.40	0.04	PA 10:0/18:2	0.31	0.00	PA 14:1/20:5	0.12	0.00
lysoPE 18:1 (sn-1)	0.47	0.04	PA 10:0/18:3	0.37	0.02	PA 14:1/21:4	0.39	0.04
lysoPE 18:2 (sn-2)	0.40	0.04	PA 11:0/22:6	0.49	0.02	PA 15:0/20:5	0.05	0.01
PA 10:0/18:1	0.38	0.01	PA 13:0/18:2	0.50	0.04	PA 15:0/25:0	0.21	0.02
PA 10:0/18:2	0.40	0.00	PA 14:1/20:5	0.10	0.00	PA 15:1/24:0	0.38	0.03
PA 10:0/18:3	0.44	0.05	PA 14:1/21:4	0.19	0.00	PA 16:0/18:3	0.34	0.01
PA 13:0/18:2	0.46	0.03	PA 15:0/18:0	0.38	0.01	PA 16:0/20:1	0.49	0.03
PA 14:1/20:5	0.12	0.00	PA 15:0/20:2	0.32	0.01	PA 16:1/18:3	0.38	0.03
PA 14:1/21:4	0.19	0.00	PA 15:0/20:5	0.07	0.00	PA 18:0/20:5	0.32	0.02
PA 15:0/18:0	0.45	0.02	PA 15:0/25:0	0.08	0.00	PA 18:3/18:4	0.50	0.04
PA 15:0/18:2	0.41	0.00	PA 15:1/24:0	0.26	0.00	PA 18:4/19:1	0.33	0.03
PA 15:0/20:2	0.46	0.04	PA 16:0/18:3	0.21	0.00	PA 19:0/22:5	0.34	0.04
PA 15:0/20:5	0.07	0.00	PA 16:0/20:1	0.48	0.02	PA 19:0/23:0	0.29	0.03
PA 15:0/25:0	0.09	0.00	PA 16:0/22:4	0.31	0.00	PA 20:0/21:5	0.23	0.01
PA 15:1/24:0	0.31	0.01	PA 16:1/18:3	0.22	0.00	PG37:1	0.39	0.05
PA 16:0/18:3	0.25	0.00	PA 17:0/13:0	0.30	0.01	PG40:6	0.46	0.02
PA 16:0/20:1	0.49	0.02	PA 17:0/18:3	0.44	0.02	PG40:8	0.42	0.02
PA 16:0/22:4	0.45	0.02	PA 17:0/20:5	0.25	0.01			
PA 16:1/18:3	0.24	0.00	PA 17:2/18:3	0.45	0.00			
PA 16:1/18:4	0.30	0.02	PA 18:0/20:1	0.42	0.00			
PA 17:0/18:3	0.42	0.02	PA 18:0/22:4	0.32	0.00			
PA 17:0/20:5	0.31	0.03	PA 18:1/18:3	0.47	0.00			
PA 17:2/18:3	0.46	0.00	PA 18:2/18:4	0.42	0.00			
PA 18:0/20:1	0.42	0.00	PA 18:3/18:4	0.26	0.00			
PA 18:0/22:4	0.32	0.00	PA 18:3/20:0	0.42	0.01			
PA 18:1/20:4	0.22	0.04	PA 18:3/20:4	0.38	0.00			
PA 18:2/18:4	0.45	0.00	PA 18:3/21:0	0.43	0.04			
PA 18:3/18:4	0.25	0.00	PA 18:3/22:4	0.30	0.00			
PA 18:3/20:0	0.47	0.02	PA 18:4/19:0	0.29	0.03			
PA 18:3/20:4	0.41	0.00	PA 18:4/19:1	0.17	0.00			
PA 18:3/21:0	0.40	0.04	PA 18:4/20:1	0.48	0.02			
PA 18:3/22:4	0.35	0.01	PA 18:4/20:2	0.21	0.00			
PA 18:4/19:0	0.28	0.03	PA 18:4/20:4	0.32	0.00			
PA 18:4/19:1	0.14	0.00	PA 18:4/20:5	0.48	0.01			

For Review Only

PA 18:4/20:2	0.34	0.02	PA 19:0/22:5	0.16	0.00			
PA 18:4/20:4	0.35	0.00	PA 19:0/23:0	0.12	0.00			
PA 18:4/20:5	0.42	0.00	PA 20:0/21:5	0.14	0.00			
PA 19:0/22:5	0.13	0.00	PA 20:4/26:2	0.35	0.00			
PA 19:0/23:0	0.12	0.01	PG 18:0/19:0	0.42	0.02			
PA 20:0/21:5	0.15	0.00	PG37:1	0.32	0.01			
PA 20:4/26:2	0.42	0.01	PG39:3	0.41	0.02			
PG 18:0/19:0	0.28	0.00	PG40:6	0.40	0.00			
PG40:8	0.44	0.03	PG40:8	0.34	0.01			
PS38:1	0.44	0.02						
PS40:1	0.27	0.02						
PS41:6	0.38	0.00						

For Review Only

Supplemental Table 8. Significantly elevated lipid elements of patients at the early and late stages of lung cancer (more than two folds), as compared with healthy control (p values)

Patient at early stage			Patients at late stage		
**Lipids**	**Folds**	**P Values**	**Lipids**	**Folds**	**P Values**
lysoPC 20:0 (sn-1)	2.32	0.03	lysoPC 17:0 (sn-1)	2.61	0.04
lysoPC 22:0 (sn-1)	2.73	0.03	lysoPC 18:0 (sn-1)	2.68	0.03
lysoPE19:0	6.36	0.01	lysoPC 19:0 (sn-1)	2.79	0.01
lysoPG15:0	5.11	0.01	lysoPC 20:0 (sn-1)	2.34	0.03
lysoPG16:1	3.14	0.00	lysoPC 20:1 (sn-1)	2.10	0.05
lysoPG18:3	2.18	0.02	lysoPC 22:0 (sn-1)	2.78	0.03
lysoPI 17:0 (sn-1)	7.39	0.04	lysoPC 22:6 (sn-1)	2.91	0.05
lysoPI 18:0 (sn-1)	3.90	0.01	lysoPG15:0	4.63	0.04
lysoPI 18:1 (sn-1)	4.44	0.01	lysoPG15:1	2.19	0.05
lysoPI 18:2 (sn-1)	3.06	0.04	lysoPG16:1	2.85	0.03
lysoPI 18:3 (sn-1)	2.95	0.03	lysoPI 22:0 (sn-1)	10.29	0.01
lysoPI 19:0 (sn-1)	13.11	0.00	lysoPS14:0	4.37	0.01
lysoPI 20:0 (sn-1)	5.71	0.00	lysoPS16:0	3.58	0.02
lysoPI 20:1 (sn-1)	3.77	0.01	lysoPS17:0	2.90	0.04
lysoPI 20:2 (sn-1)	11.62	0.00	lysoPS17:1	12.30	0.03
lysoPI 20:3 (sn-1)	6.60	0.01	lysoPS18:1	3.36	0.04
lysoPI 20:4 (sn-1)	3.55	0.02	lysoPS18:2	5.60	0.02
lysoPI 22:0 (sn-1)	11.01	0.02	lysoPS18:3	4.89	0.01
lysoPI 22:4 (sn-1)	4.75	0.00	lysoPS20:2	3.30	0.04
lysoPI 22:6 (sn-1)	8.18	0.00	lysoPS20:5	2.74	0.05
lysoPS14:0	3.29	0.02	lysoPS22:6	3.37	0.04
lysoPS15:0	2.54	0.02	PC 37:3; PC 17:0/20:3 or 19:1/18:2	2.09	0.03
lysoPS15:1	3.71	0.01	PC 37:4; PC 17:0/20:4	2.17	0.02
lysoPS16:0	5.59	0.04	PC 37:5e; PC 16:0e/22:5 or 18:0e/20:5	2.07	0.01
lysoPS17:0	3.13	0.02	PC 39:0 (18:0/21:0)	2.07	0.00
lysoPS17:1	7.37	0.03	PC 39:2 (18:0/21:2)	2.53	0.05
lysoPS18:2	3.10	0.01	PC 39:3 (18:0/21:3)	3.52	0.03
lysoPS20:3	2.08	0.01	PC 39:3; PC 19:0/20:3	3.53	0.03
lysoPS20:5	2.92	0.02	PC 39:4 (18:0/21:4)	3.53	0.03
lysoPS22:0	2.98	0.00	PC 39:5 (18:0/21:5)	3.10	0.03
PC 19:0/19:0	2.32	0.03	PC 39:6; PC 17:0/22:6	3.18	0.01
PC 19:0/21:2	2.46	0.04	PC 39:7; PC 17:1/22:6	2.72	0.00
PE 32:2; PE 14:0/18:2	3.42	0.03	PC 40:1; PC 18:1/22:0	2.47	0.03
PE 35:2; PE 17:0/18:2	2.37	0.02	PC 40:4; PC 18:0/22:4 or 20:1/20:3	2.44	0.00
PE 35:6p; PE 16:0p/20:5	2.27	0.02	PC 40:5; PC 18:0/22:5	2.22	0.00
PE 36:1; PE 16:0/20:1 or 18:0/18:1	2.01	0.03	PC 40:6; PC 18:0/22:6	2.08	0.00
PE 36:5; PE 16:0/20:5	2.05	0.04	PC 41:6; PC 19:0/22:6	4.93	0.03
PE 37:3; PE 17:0/20:3	2.55	0.03	PC 42:5	2.87	0.01
PE 38:1; PE 18:0/20:1	2.40	0.01	PE 35:5p; PE 16:0p/20:4	2.47	0.03
PE 38:2; PE 18:1/20:1	2.14	0.04	PE 36:1; PE 16:0/20:1 or 18:0/18:1	2.61	0.01

For Review Only

PE 39:7; PE 17:1/22:6	2.35	0.01	PE 37:6p; PE 18:0p/20:5 or 18:1p/20:4 or 16:0e/22:6	2.66	0.03
PE 40:1; PE 22:0/18:1	2.71	0.00	PE 37:7p; PE 16:0p/22:6	2.89	0.03
PE 40:2; PE 18:1/22:1	2.68	0.01	PE 38:1; PE 18:0/20:1	4.01	0.01
PE 40:3; PE 18:1/22:2 or 22:1/18:2	2.59	0.01	PE 38:2; PE 18:1/20:1	3.83	0.01
PG30:0	2.39	0.03	PE 38:3; PE 18:0/20:3	3.33	0.01
PG30:1	4.16	0.00	PE 39:7; PE 17:1/22:6	3.70	0.01
PG31:0	2.45	0.00	PE 40:2; PE 18:1/22:1	4.61	0.00
PG31:1	2.83	0.01	PE 40:3; PE 18:1/22:2 or 22:1/18:2	4.82	0.00
PG32:0	2.48	0.03	PE 40:4; PE 18:0/22:4 or 20:0/20:4	3.24	0.01
PG32:2	2.03	0.04	PE 40:7; PE 18:1/22:6	3.85	0.02
PG33:2	2.10	0.01	PE 42:8; PE 20:2/22:6	4.29	0.05
PG34:4	2.44	0.02	PG30:0	5.38	0.00
PG34:5	2.54	0.03	PG30:1	6.61	0.00
PI 30:1	2.03	0.04	PG31:0	4.91	0.00
PI 31:1p; PI 16:0p/16:0	2.19	0.02	PG31:1	5.25	0.00
PI 36:3; PI 16:0/20:3 or 18:0/18:3 or 18:1/18:2	8.01	0.00	PG32:0	3.70	0.00
PS30:0	4.72	0.00	PG32:1	3.05	0.00
PS30:1	9.71	0.00	PG32:2	3.16	0.00
PS31:0	3.61	0.00	PG33:0	2.69	0.00
PS31:1	5.57	0.00	PG33:1	2.64	0.00
PS32:1	7.67	0.03	PG34:0	3.99	0.00
PS33:0	2.26	0.01	PG34:1	4.07	0.00
PS33:2	2.50	0.01	PG34:2	2.19	0.00
PS34:3	2.62	0.00	PG34:4	3.24	0.00
PS34:4	2.54	0.00	PG34:5	3.69	0.00
PS34:5	2.65	0.00	PG35:4	2.00	0.03
PS35:3	2.07	0.02	PG36:1	2.15	0.01
PS35:5	2.62	0.03	PG36:2	2.05	0.02
PS36:5	3.75	0.00	PS30:0	7.76	0.02
PS36:6	3.17	0.00	PS30:1	12.96	0.00
PS37:3	2.43	0.00	PS31:0	5.83	0.00
PS37:6	2.76	0.00	PS31:1	5.32	0.01
			PS32:0	3.20	0.02
			PS32:1	4.45	0.02
			PS34:2	2.94	0.02
			PS34:3	3.20	0.00
			PS34:4	3.45	0.00
			PS34:5	3.27	0.00
			PS35:2	2.02	0.01
			PS35:3	2.85	0.00
			PS36:5	3.25	0.04
			PS36:6	3.04	0.02
			PS37:2	3.68	0.05
			PS37:3	2.62	0.02
			PS37:6	3.72	0.00
			PS38:7	2.98	0.02

For Review Only

Supplemental Table 9. Significantly declined lipid elements of patients at the early and late stages of lung cancer (more than two folds), as compared with healthy control (p values)

Patient at early stage			Patients at late stage		
**Lipids**	**Folds**	**P Values**	**Lipids**	**Folds**	**P Values**
PA 14:1/20:5	0.09	0.00	lysoPS20:0	0.38	0.01
PA 14:1/21:4	0.29	0.04	PA 10:0/18:1	0.36	0.00
PA 15:0/20:5	0.07	0.02	PA 10:0/18:2	0.36	0.00
PA 15:0/25:0	0.23	0.05	PA 10:0/18:3	0.48	0.01
PA 16:0/18:3	0.34	0.02	PA 14:1/20:5	0.12	0.00
PA 16:1/18:3	0.34	0.04	PA 14:1/21:4	0.24	0.00
PA 18:4/19:1	0.26	0.04	PA 15:0/18:0	0.43	0.00
PA 20:0/21:5	0.28	0.04	PA 15:0/18:2	0.49	0.00
PG40:6	0.50	0.05	PA 15:0/20:2	0.43	0.00
PG40:8	0.38	0.03	PA 15:0/20:5	0.06	0.00
			PA 15:0/25:0	0.09	0.00
			PA 15:1/24:0	0.26	0.00
			PA 16:0/18:3	0.24	0.00
			PA 16:0/20:1	0.44	0.00
			PA 16:0/22:4	0.43	0.00
			PA 16:1/18:3	0.26	0.00
			PA 17:0/13:0	0.43	0.00
			PA 17:0/20:5	0.31	0.00
			PA 18:0/20:1	0.44	0.00
			PA 18:0/22:4	0.37	0.00
			PA 18:1/20:4	0.25	0.00
			PA 18:2/18:4	0.49	0.00
			PA 18:2/20:4	0.26	0.01
			PA 18:3/18:4	0.28	0.00
			PA 18:3/20:4	0.44	0.00
			PA 18:3/21:0	0.45	0.01
			PA 18:3/22:4	0.36	0.00
			PA 18:4/19:0	0.30	0.00
			PA 18:4/19:1	0.19	0.00
			PA 18:4/20:2	0.34	0.00
			PA 18:4/20:4	0.44	0.00
			PA 18:4/20:5	0.45	0.00
			PA 19:0/22:5	0.14	0.00
			PA 19:0/23:0	0.13	0.00
			PA 20:0/21:5	0.14	0.00
			PA 20:4/26:2	0.41	0.00
			PG 18:0/19:0	0.34	0.00
			PG37:1	0.46	0.02
			PG40:6	0.44	0.00
			PG40:8	0.40	0.00
			PS40:1	0.37	0.00
			PS41:6	0.47	0.00

For Review Only

Supplemental Table 10. Significantly elevated lipid elements of lung cancer patients with or without metastasis (more than two folds), as compared with healthy control (p values)

Patients without metastasis			Patients with metastasis		
**Lipids**	**Folds**	**P values**	**Lipids**	**Folds**	**P values**
C1P160 Mean	3.88	0.00	lysoPC 17:0 (sn-1)	2.96	0.03
C1P240 Mean	5.38	0.01	lysoPC 18:0 (sn-1)	2.97	0.02
lysoPE19:0	5.41	0.03	lysoPC 19:0 (sn-1)	2.99	0.01
lysoPG14:0	8.59	0.00	lysoPC 20:0 (sn-1)	2.55	0.02
lysoPG15:0	6.94	0.00	lysoPC 22:0 (sn-1)	3.03	0.02
lysoPG16:1	3.30	0.01	lysoPC 22:6 (sn-1)	3.20	0.04
lysoPG22:6	2.22	0.02	lysoPG15:1	2.27	0.04
lysoPI 18:0 (sn-1)	4.16	0.02	lysoPG16:1	2.77	0.03
lysoPI 19:0 (sn-1)	5.68	0.01	lysoPI 22:0 (sn-1)	11.39	0.01
lysoPI 20:0 (sn-1)	4.91	0.01	lysoPS14:0	4.09	0.01
lysoPI 20:1 (sn-1)	3.56	0.01	lysoPS15:0	2.46	0.04
lysoPI 20:2 (sn-1)	4.97	0.01	lysoPS16:0	3.68	0.02
lysoPI 20:3 (sn-1)	4.84	0.03	lysoPS17:0	2.95	0.04
lysoPI 20:4 (sn-1)	3.20	0.03	lysoPS17:1	11.04	0.04
lysoPI 22:4 (sn-1)	5.00	0.00	lysoPS18:1	3.25	0.04
lysoPI 22:6 (sn-1)	6.67	0.01	lysoPS18:2	5.21	0.01
lysoPS14:0	4.32	0.03	lysoPS18:3	4.63	0.01
lysoPS15:1	4.55	0.02	lysoPS22:0	2.74	0.04
lysoPS17:0	2.92	0.05	lysoPS22:6	2.97	0.03
lysoPS18:3	5.71	0.05	PC 37:3;PC 17:0/20:3 or 19:1/18:2	2.25	0.02
lysoPS19:0	2.85	0.04	PC 37:4; PC 17:0/20:4	2.30	0.02
lysoPS20:2	3.24	0.04	PC 37:5e;PC 16:0e/22:5 or 18:0e/20:5	2.21	0.01
lysoPS20:3	2.21	0.02	PC 39:0 (18:0/21:0)	2.09	0.00
lysoPS20:5	3.16	0.01	PC 39:2 (18:0/21:2)	2.82	0.04
PC 18:1/23:1	3.27	0.00	PC 39:3 (18:0/21:3)	3.88	0.03
PC 41:6; PC 19:0/22:6	2.24	0.01	PC 39:3; PC 19:0/20:3	3.85	0.03
PE 37:3; PE 17:0/20:3	2.31	0.05	PC 39:4 (18:0/21:4)	3.89	0.03
PE 38:1; PE 18:0/20:1	2.30	0.04	PC 39:5 (18:0/21:5)	3.37	0.03
PE 39:7; PE 17:1/22:6	2.05	0.05	PC 39:6; PC 17:0/22:6	3.48	0.01
PE 40:2; PE 18:1/22:1	2.44	0.03	PC 39:7; PC 17:1/22:6	2.95	0.00
PG30:1	4.89	0.02	PC 40:1; PC 18:1/22:0	2.74	0.02
PG31:0	3.31	0.02	PC 40:4; PC 18:0/22:4 or 20:1/20:3	2.64	0.00
PG31:1	3.84	0.04	PC 40:5; PC 18:0/22:5	2.30	0.00
PG34:4	2.72	0.00	PC 40:6; PC 18:0/22:6	2.07	0.00
PG34:5	3.29	0.01	PC 40:8; PC 20:4/20:4	2.03	0.02
PG36:6	2.66	0.01	PC 41:6; PC 19:0/22:6	5.41	0.03
PI 36:3; PI 16:0/20:3 or 18:0/18:3 or 18:1/18:2	7.10	0.00	PC 42:5	3.19	0.01
PS30:1	11.89	0.00	PE 35:5p; PE 16:0p/20:4	2.57	0.02
PS31:0	5.29	0.00	PE 35:6p; PE 16:0p/20:5	2.60	0.04
PS31:1	6.10	0.01	PE 36:1; PE 16:0/20:1 or 18:0/18:1	2.74	0.01
PS32:1	5.56	0.05	PE 37:6p; PE 18:0p/20:5 or 18:1p/20:4 or 16:0e/22:6	2.83	0.03
PS33:0	2.52	0.02	PE 37:7p; PE 16:0p/22:6	3.06	0.03

For Review Only

PS34:3	2.17	0.00	PE 38:1; PE 18:0/20:1	4.17	0.01
PS34:4	2.54	0.01	PE 38:2; PE 18:1/20:1	3.91	0.01
PS34:5	2.43	0.01	PE 38:3; PE 18:0/20:3	3.37	0.01
PS35:5	5.20	0.04	PE 38:6; PE 16:0/22:6 or 16:1/22:5 or 20:2/18:4	2.76	0.05
PS36:5	3.60	0.01	PE 39:7; PE 17:1/22:6	3.90	0.01
PS36:6	3.05	0.00	PE 40:2; PE 18:1/22:1	4.84	0.00
PS37:3	3.34	0.01	PE 40:3; PE 18:1/22:2 or 22:1/18:2	5.03	0.00
PS37:6	3.27	0.00	PE 40:4; PE 18:0/22:4 or 20:0/20:4	3.32	0.01
SM d18:0/26:1 +HN4+	2.68	0.02	PE 40:7; PE 18:1/22:6	4.14	0.01
TAG 53:2	2.90	0.01	PE 42:8; PE 20:2/22:6	4.64	0.04
TAG 53:3	2.85	0.01	PG30:0	5.06	0.00
TAG 56:5	8.09	0.00	PG30:1	6.54	0.00
			PG31:0	4.79	0.00
			PG31:1	5.08	0.00
			PG32:0	3.66	0.00
			PG32:1	3.06	0.00
			PG32:2	3.13	0.00
			PG33:0	2.73	0.00
			PG33:1	2.65	0.00
			PG34:0	3.96	0.00
			PG34:1	4.06	0.00
			PG34:2	2.14	0.00
			PG34:4	3.20	0.00
			PG34:5	3.51	0.01
			PG35:4	2.06	0.02
			PG36:1	2.29	0.01
			PG36:2	2.24	0.02
			PS30:0	6.53	0.00
			PS30:1	12.44	0.00
			PS31:0	5.40	0.01
			PS31:1	5.12	0.01
			PS32:0	3.23	0.02
			PS32:1	4.95	0.04
			PS33:2	2.13	0.01
			PS34:2	2.68	0.01
			PS34:3	3.40	0.00
			PS34:4	3.51	0.00
			PS34:5	3.39	0.00
			PS35:1	2.08	0.01
			PS35:2	2.02	0.01
			PS35:3	2.79	0.00
			PS36:5	3.27	0.04
			PS36:6	3.07	0.03
			PS37:2	3.16	0.04
			PS37:3	2.31	0.02
			PS37:6	3.61	0.00
			PS38:7	3.38	0.02

For Review Only

Supplemental Table 11. Significantly declined lipid elements of lung cancer patients with or without metastasis (more than two folds), as compared with healthy control (p values)

Patients without metastasis			Patients with metastasis		
**Lipids**	**Folds**	**P values**	**Lipids**	**Folds**	**P values**
PA 10:0/18:2	0.43	0.02	lysoPS20:0	0.39	0.02
PA 14:1/20:5	0.12	0.00	PA 10:0/18:1	0.39	0.00
PA 14:1/21:4	0.31	0.03	PA 10:0/18:2	0.38	0.00
PA 15:0/20:5	0.08	0.01	PA 14:1/20:5	0.11	0.00
PA 15:0/25:0	0.20	0.02	PA 14:1/21:4	0.23	0.00
PA 15:1/24:0	0.40	0.04	PA 15:0/18:0	0.45	0.00
PA 16:0/18:3	0.32	0.01	PA 15:0/18:2	0.49	0.00
PA 16:0/18:4	0.29	0.04	PA 15:0/20:2	0.46	0.01
PA 16:1/18:3	0.34	0.03	PA 15:0/20:5	0.06	0.00
PA 18:3/18:4	0.43	0.03	PA 15:0/25:0	0.10	0.00
PA 18:4/19:1	0.21	0.02	PA 15:1/24:0	0.29	0.00
PA 19:0/23:0	0.29	0.04	PA 16:0/18:3	0.24	0.00
PA 20:0/21:5	0.24	0.02	PA 16:0/20:1	0.43	0.00
PG40:6	0.49	0.04	PA 16:0/22:4	0.46	0.00
PG40:8	0.40	0.03	PA 16:1/18:3	0.25	0.00
			PA 17:0/13:0	0.41	0.00
			PA 17:0/20:5	0.33	0.00
			PA 18:0/20:1	0.46	0.00
			PA 18:0/22:4	0.40	0.00
			PA 18:1/20:4	0.26	0.00
			PA 18:2/20:4	0.26	0.01
			PA 18:3/18:4	0.29	0.00
			PA 18:3/20:4	0.46	0.00
			PA 18:3/21:0	0.46	0.01
			PA 18:3/22:4	0.39	0.00
			PA 18:4/19:0	0.31	0.00
			PA 18:4/19:1	0.20	0.00
			PA 18:4/20:2	0.38	0.00
			PA 18:4/20:4	0.47	0.00
			PA 18:4/20:5	0.44	0.00
			PA 19:0/22:5	0.14	0.00
			PA 19:0/23:0	0.13	0.00
			PA 20:0/21:5	0.14	0.00
			PA 20:4/26:2	0.45	0.00
			PG 18:0/19:0	0.34	0.00
			PG37:1	0.48	0.04
			PG40:6	0.44	0.00
			PG40:8	0.40	0.00
			PS40:1	0.40	0.01
			PS41:6	0.42	0.00

For Review Only

Supplemental Table 12. Significantly elevated lipid elements of lung cancer patients with body mass index (BMI) less or more than 22, as compared with healthy control (more than two folds, p values).

**Patient BMI <22**			**Patients BMI >22**			
**Lipids**	**Folds**	**P values**	**Lipids**		**Folds**	**P values**
lysoPC 14:0 (sn-2)	3.46	0.04	lysoPC 22:0 (sn-1)		2.48	0.04
lysoPC 15:0 (sn-2)	2.77	0.05	lysoPG15:0		3.96	0.04
lysoPC 15:1 (sn-1)	4.72	0.04	lysoPG16:1		2.35	0.04
lysoPC 16:0 (sn-2)	2.75	0.03	lysoPI 18:0 (sn-1)		4.99	0.03
lysoPC 16:1 (sn-1)	3.99	0.02	lysoPI 18:1 (sn-1)		5.48	0.03
lysoPC 17:0 (sn-1)	2.88	0.02	lysoPI 19:0 (sn-1)		7.22	0.03
lysoPC 17:1 (sn-1)	2.91	0.03	lysoPI 20:0 (sn-1)		3.89	0.02
lysoPC 18:0 (sn-1)	2.86	0.01	lysoPI 20:2 (sn-1)		7.44	0.03
lysoPC 18:1 (sn-1)	2.89	0.03	lysoPI 20:3 (sn-1)		5.11	0.01
lysoPC 18:3 (sn-1)	4.40	0.03	lysoPI 20:4 (sn-1)		2.85	0.04
lysoPC 19:0 (sn-1)	3.18	0.00	lysoPI 22:0 (sn-1)		11.23	0.00
lysoPC 20:0 (sn-1)	2.71	0.01	lysoPI 22:4 (sn-1)		5.53	0.00
lysoPC 20:1 (sn-1)	2.25	0.04	lysoPI 22:6 (sn-1)		6.81	0.01
lysoPC 20:2 (sn-1)	2.37	0.02	lysoPS14:0		4.39	0.01
lysoPC 20:3 (sn-1)	2.61	0.02	lysoPS15:1		4.46	0.01
lysoPC 20:4 (sn-1)	3.12	0.03	lysoPS16:0		5.07	0.02
lysoPC 22:0 (sn-1)	3.09	0.03	lysoPS17:0		3.07	0.02
lysoPC 22:6 (sn-1)	2.95	0.02	lysoPS17:1		11.53	0.04
lysoPG15:0	5.56	0.03	lysoPS18:2		4.77	0.03
lysoPG16:1	3.51	0.01	lysoPS18:3		4.95	0.03
lysoPI 22:0 (sn-1)	9.59	0.03	lysoPS20:1		2.28	0.03
lysoPS14:0	3.88	0.04	lysoPS20:2		2.74	0.04
lysoPS15:0	2.44	0.03	lysoPS20:5		2.88	0.02
lysoPS16:0	2.83	0.03	PC 39:6; PC 17:0/22:6		2.58	0.04
lysoPS17:1	11.04	0.05	PC 39:7; PC 17:1/22:6		2.29	0.01
lysoPS18:1	2.99	0.04	PE 36:1; PE 16:0/20:1 or 18:0/18:1		2.00	0.03
lysoPS18:2	5.45	0.03	PE 38:1; PE 18:0/20:1		2.70	0.01
lysoPS18:3	4.87	0.01	PE 38:2; PE 18:1/20:1		2.65	0.04
lysoPS22:4	2.45	0.02	PE 38:3; PE 18:0/20:3		2.47	0.04
lysoPS22:6	3.05	0.02	PE 39:7; PE 17:1/22:6		2.34	0.01
PC 18:1/23:1	11.58	0.05	PE 40:1; PE 22:0/18:1		2.50	0.01
PC 37:3; PC 17:0/20:3 or 19:1/18:2	2.23	0.03	PE 40:2; PE 18:1/22:1		3.09	0.00
PC 37:4; PC 17:0/20:4	2.36	0.02	PE 40:3; PE 18:1/22:2 22:1/18:2		3.30	0.01
PC 37:5e; PC 16:0e/22:5 or 18:0e/20:5	2.29	0.01	PE 40:4; PE 18:0/22:4 or 20:0/20:4		2.32	0.03
PC 39:0 (18:0/21:0)	2.21	0.00	PE 40:7; PE 18:1/22:6		2.19	0.04
PC 39:3 (18:0/21:3)	3.80	0.03	PE 42:8; PE 20:2/22:6		2.33	0.04
PC 39:3; PC 19:0/20:3	3.81	0.03	PG30:0		4.48	0.01
PC 39:4 (18:0/21:4)	4.02	0.03	PG30:1		5.44	0.00
PC 39:5 (18:0/21:5)	3.44	0.03	PG31:0		4.20	0.00
PC 39:6; PC 17:0/22:6	3.45	0.01	PG31:1		4.95	0.00
PC 39:7; PC 17:1/22:6	2.88	0.01	PG32:0		3.26	0.00
PC 40:1; PC 18:1/22:0	2.54	0.02	PG32:1		2.91	0.00
PC 40:4; PC 18:0/22:4 or 20:1/20:3	2.77	0.00	PG32:2		2.85	0.00
PC 40:5; PC 18:0/22:5	2.48	0.00	PG33:0		2.49	0.01
PC 40:6; PC 18:0/22:6	2.22	0.00	PG33:1		2.48	0.00
PC 40:7; PC 18:1/22:6	2.09	0.00	PG34:0		3.41	0.00
PC 41:6; PC 19:0/22:6	5.16	0.02	PG34:1		3.66	0.01

For Review Only

PC 42:5	3.10	0.02	PG34:2		2.12	0.01
PE 35:5p; PE 16:0p/20:4	2.80	0.03	PG34:4		2.75	0.00
PE 35:6p; PE 16:0p/20:5	2.88	0.04	PG34:5		3.14	0.00
PE 36:1; PE 16:0/20:1 or 18:0/18:1	3.01	0.01	PI 30:1		2.13	0.01
PE 37:6p; PE 18:0p/20:5 or 18:1p/20:4or 16:0e/22:6	3.21	0.02	PI 31:1p; PI 16:0p/16:0		2.36	0.00
PE 37:7p; PE 16:0p/22:6	3.55	0.02	PI 36:3; PI 16:0/20:3 or 18:0/18:3 or 18:1/18:2		11.12	0.00
PE 38:1; PE 18:0/20:1	4.74	0.01	PS30:0		7.61	0.05
PE 38:2; PE 18:1/20:1	4.39	0.01	PS30:1		11.33	0.00
PE 38:3; PE 18:0/20:3	3.67	0.01	PS31:0		4.86	0.00
PE 38:6; PE 16:0/22:6 or 16:1/22:5 or 20:2/18:4	3.25	0.04	PS31:1		5.48	0.01
PE 39:7; PE 17:1/22:6	4.59	0.01	PS32:0		2.92	0.04
PE 40:2; PE 18:1/22:1	5.43	0.00	PS32:1		6.21	0.03
PE 40:3; PE 18:1/22:2 or 22:1/18:2	5.52	0.00	PS33:2		2.13	0.04
PE 40:4; PE 18:0/22:4 or 20:0/20:4	3.60	0.01	PS34:3		2.59	0.00
PE 40:6; PE 18:0/22:6	4.21	0.05	PS34:4		2.71	0.00
PE 40:7; PE 18:1/22:6	4.94	0.01	PS34:5		2.61	0.00
PE 42:8; PE 20:2/22:6	5.42	0.04	PS35:1		2.11	0.02
PG30:0	5.07	0.00	PS35:2		2.16	0.02
PG30:1	6.84	0.00	PS35:3		2.26	0.04
PG31:0	4.62	0.00	PS36:5		3.65	0.02
PG31:1	4.55	0.00	PS36:6		3.27	0.02
PG32:0	3.67	0.00	PS37:3		2.82	0.01
PG32:1	2.93	0.00	PS37:6		3.26	0.00
PG32:2	3.02	0.00	PS38:7		3.36	0.05
PG33:0	2.61	0.00				
PG33:1	2.51	0.00				
PG34:0	3.88	0.00				
PG34:1	3.80	0.00				
PG34:2	2.15	0.00				
PG34:4	3.43	0.01				
PG34:5	3.79	0.01				
PG35:4	2.12	0.04				
PG36:1	2.30	0.01				
PG36:2	2.26	0.02				
PS30:0	6.65	0.01				
PS30:1	13.35	0.00				
PS31:0	5.93	0.01				
PS31:1	5.25	0.00				
PS32:0	2.84	0.04				
PS32:1	3.92	0.02				
PS34:2	2.70	0.02				
PS34:3	3.60	0.00				
PS34:4	3.86	0.00				
PS34:5	3.72	0.00				
PS35:3	3.16	0.00				
PS36:5	3.04	0.04				
PS36:6	2.84	0.02				
PS37:3	2.32	0.04				
PS37:6	3.80	0.00				
PS38:7	2.84	0.02				

For Review Only

Supplemental Table 13. Significantly declined lipid elements of lung cancer patients with body mass index (BMI) less or more than 22, as compared with healthy control (more than two folds, p values).

**Patient BMI≤22**			**Patients BMI >22**		
**Lipids**	**Folds**	**P values**	**Lipids**	**Folds**	**P values**
PA 10:0/18:1	0.45	0.02	lysoPS20:0	0.36	0.04
PA 10:0/18:2	0.33	0.00	PA 10:0/18:1	0.45	0.01
PA 10:0/18:3	0.41	0.01	PA 10:0/18:2	0.45	0.00
PA 14:1/20:5	0.10	0.00	PA 14:1/20:5	0.13	0.00
PA 14:1/21:4	0.22	0.00	PA 14:1/21:4	0.27	0.00
PA 15:0/18:0	0.49	0.02	PA 15:0/18:0	0.50	0.01
PA 15:0/18:2	0.50	0.00	PA 15:0/20:5	0.08	0.00
PA 15:0/20:2	0.45	0.02	PA 15:0/25:0	0.15	0.00
PA 15:0/20:5	0.04	0.00	PA 15:1/24:0	0.36	0.00
PA 15:0/25:0	0.09	0.00	PA 16:0/18:3	0.28	0.00
PA 15:1/24:0	0.27	0.00	PA 16:0/22:4	0.48	0.01
PA 16:0/18:3	0.25	0.00	PA 16:1/18:3	0.30	0.00
PA 16:0/18:4	0.21	0.00	PA 17:0/13:0	0.38	0.01
PA 16:0/20:1	0.43	0.00	PA 17:0/20:5	0.31	0.01
PA 16:0/22:4	0.49	0.01	PA 18:1/20:4	0.31	0.02
PA 16:1/18:3	0.24	0.00	PA 18:2/20:4	0.29	0.05
PA 16:1/18:4	0.24	0.00	PA 18:3/18:4	0.36	0.00
PA 17:0/20:5	0.34	0.01	PA 18:3/22:4	0.48	0.01
PA 17:1/19:0	0.50	0.02	PA 18:4/19:0	0.39	0.02
PA 17:2/18:3	0.47	0.00	PA 18:4/19:1	0.21	0.00
PA 18:0/20:1	0.43	0.00	PA 18:4/20:2	0.49	0.05
PA 18:0/22:4	0.35	0.00	PA 19:0/22:5	0.24	0.00
PA 18:1/20:4	0.24	0.01	PA 19:0/23:0	0.21	0.00
PA 18:3/18:4	0.29	0.00	PA 20:0/21:5	0.19	0.00
PA 18:3/20:4	0.44	0.00	PG37:1	0.34	0.01
PA 18:3/21:0	0.46	0.03	PG39:3	0.43	0.01
PA 18:3/22:4	0.36	0.00	PG40:6	0.42	0.00
PA 18:4/19:0	0.31	0.01	PG40:8	0.36	0.00
PA 18:4/19:1	0.20	0.00	PS40:1	0.43	0.04
PA 18:4/20:2	0.33	0.01			
PA 18:4/20:5	0.44	0.00			
PA 19:0/22:5	0.16	0.00			
PA 19:0/23:0	0.14	0.00			
PA 20:0/21:5	0.15	0.00			
PA 20:4/26:2	0.43	0.00			
PG 18:0/19:0	0.41	0.01			
PG40:6	0.49	0.01			
PG40:8	0.44	0.01			
PS38:1	0.47	0.01			
PS40:1	0.36	0.02			
PS41:6	0.42	0.00			

For Review Only

Supplemental Table 14. Significantly elevated lipid elements of lung cancer patients with digital evaluation score system (DESS) scores less or more than 90, as compared with healthy control (more than two folds, p values).

Patient DESS scores <90			Patient DESS scores >90		
**Lipids**	**Folds**	**P values**	**Lipids**	**Folds**	**P values**
lysoPC 15:1 (sn-1)	3.18	0.04	lysoPC 19:0 (sn-1)	2.70	0.01
lysoPC 17:0 (sn-1)	2.56	0.03	lysoPC 20:0 (sn-1)	2.22	0.04
lysoPC 17:1 (sn-1)	2.39	0.04	lysoPC 22:0 (sn-1)	2.55	0.04
lysoPC 18:0 (sn-1)	2.77	0.02	lysoPG15:0	4.25	0.02
lysoPC 18:1 (sn-1)	2.63	0.05	lysoPG16:1	2.88	0.01
lysoPC 19:0 (sn-1)	2.64	0.01	lysoPI 22:0 (sn-1)	9.95	0.03
lysoPC 20:0 (sn-1)	2.46	0.03	lysoPS14:0	5.13	0.01
lysoPC 20:2 (sn-1)	2.35	0.05	lysoPS16:0	3.79	0.04
lysoPC 20:3 (sn-1)	2.37	0.03	lysoPS17:0	3.41	0.03
lysoPC 20:4 (sn-1)	2.74	0.05	lysoPS17:1	13.68	0.04
lysoPC 22:0 (sn-1)	2.99	0.03	lysoPS18:1	4.07	0.04
lysoPC 22:6 (sn-1)	2.80	0.04	lysoPS18:2	7.02	0.01
lysoPG14:0	6.44	0.00	lysoPS18:3	5.12	0.00
lysoPG15:0	5.20	0.04	lysoPS20:1	2.69	0.05
lysoPG16:1	2.94	0.04	lysoPS20:2	4.06	0.03
lysoPI 18:0 (sn-1)	4.59	0.03	lysoPS20:5	3.36	0.03
lysoPI 18:1 (sn-1)	4.28	0.01	lysoPS22:0	3.66	0.04
lysoPI 19:0 (sn-1)	7.96	0.02	lysoPS22:6	4.08	0.03
lysoPI 20:0 (sn-1)	3.95	0.02	PC 39:3 (18:0/21:3)	3.73	0.05
lysoPI 20:2 (sn-1)	8.24	0.02	PC 39:3; PC 19:0/20:3	3.81	0.05
lysoPI 20:3 (sn-1)	5.09	0.01	PC 39:6; PC 17:0/22:6	3.27	0.03
lysoPI 22:0 (sn-1)	10.93	0.01	PC 39:7; PC 17:1/22:6	2.69	0.02
lysoPI 22:4 (sn-1)	5.31	0.00	PC 40:4; PC 18:0/22:4 or 20:1/20:3	2.25	0.02
lysoPI 22:6 (sn-1)	7.36	0.00	PC 40:5; PC 18:0/22:5	2.05	0.00
lysoPS14:0	3.17	0.01	PC 42:5	3.03	0.02
lysoPS15:0	2.23	0.03	PE 36:1; PE 16:0/20:1 or 18:0/18:1	2.71	0.03
lysoPS16:0	4.19	0.04	PE 38:1; PE 18:0/20:1	4.12	0.02
lysoPS17:1	8.91	0.04	PE 38:2; PE 18:1/20:1	3.92	0.02
lysoPS18:1	2.46	0.04	PE 38:3; PE 18:0/20:3	3.22	0.01
lysoPS18:2	3.17	0.00	PE 39:7; PE 17:1/22:6	3.89	0.02
lysoPS22:0	2.12	0.03	PE 40:2; PE 18:1/22:1	4.78	0.01
PC 39:0 (18:0/21:0)	2.16	0.00	PE 40:3; PE 18:1/22:2 or 22:1/18:2	4.87	0.01
PC 39:3 (18:0/21:3)	2.91	0.04	PE 40:4; PE 18:0/22:4 or 20:0/20:4	3.21	0.02
PC 39:3; PC 19:0/20:3	2.86	0.03	PE 40:7; PE 18:1/22:6	3.98	0.04
PC 39:4 (18:0/21:4)	2.87	0.01	PG30:0	5.70	0.00
PC 39:5 (18:0/21:5)	2.54	0.02	PG30:1	6.83	0.00
PC 39:6; PC 17:0/22:6	2.72	0.01	PG31:0	5.17	0.00
PC 39:7; PC 17:1/22:6	2.46	0.00	PG31:1	5.48	0.00
PC 40:1; PC 18:1/22:0	2.07	0.04	PG32:0	3.80	0.00
PC 40:4; PC 18:0/22:4 or 20:1/20:3	2.40	0.00	PG32:1	3.02	0.00
PC 40:5; PC 18:0/22:5	2.14	0.00	PG32:2	3.29	0.00
PC 40:7; PC 18:1/22:6	2.01	0.00	PG33:0	2.63	0.00
PC 41:6; PC 19:0/22:6	3.76	0.02	PG33:1	2.56	0.00
PE 35:5p; PE 16:0p/20:4	2.15	0.02	PG34:0	3.97	0.00
PE 35:6p; PE 16:0p/20:5	2.26	0.02	PG34:1	4.21	0.00
PE 36:1; PE 16:0/20:1 or 18:0/18:1	2.26	0.01	PG34:2	2.23	0.01
PE 36:5; PE 16:0/20:5	2.31	0.03	PG34:4	3.40	0.01

For Review Only

PE 37:6p; PE 18:0p/20:5 or 18:1p/20:4 or 16:0e/22:6	2.41	0.03	PG34:5	3.65	0.01
PE 37:7p; PE 16:0p/22:6	2.36	0.02	PG36:1	2.21	0.01
PE 38:1; PE 18:0/20:1	3.25	0.00	PG36:2	2.07	0.02
PE 38:2; PE 18:1/20:1	3.05	0.02	PS30:0	9.05	0.03
PE 38:3; PE 18:0/20:3	2.87	0.02	PS30:1	13.37	0.00
PE 38:5; PE 18:0/20:5	2.40	0.01	PS31:0	6.57	0.00
PE 38:7; PE 16:1/22:6 or 18:2/20:5	2.52	0.02	PS31:1	6.87	0.00
PE 39:7; PE 17:1/22:6	2.96	0.01	PS32:0	3.58	0.02
PE 40:1; PE 22:0/18:1	3.34	0.01	PS32:1	4.91	0.03
PE 40:2; PE 18:1/22:1	3.65	0.00	PS33:2	2.05	0.03
PE 40:3; PE 18:1/22:2 or 22:1/18:2	3.87	0.00	PS34:2	3.39	0.03
PE 40:4; PE 18:0/22:4 or 20:0/20:4	2.66	0.02	PS34:3	3.19	0.00
PE 40:7; PE 18:1/22:6	3.04	0.02	PS34:4	3.63	0.00
PE 42:8; PE 20:2/22:6	3.00	0.03	PS34:5	3.50	0.00
PG30:0	3.84	0.01	PS35:2	2.11	0.02
PG30:1	5.39	0.00	PS35:3	3.20	0.00
PG31:0	3.64	0.00	PS36:5	4.02	0.02
PG31:1	4.04	0.00	PS36:6	3.64	0.02
PG32:0	3.10	0.00	PS37:2	4.74	0.03
PG32:1	2.81	0.00	PS37:3	3.16	0.01
PG32:2	2.57	0.00	PS37:5	2.05	0.03
PG33:0	2.47	0.01	PS37:6	3.93	0.00
PG33:1	2.43	0.00	PS38:7	3.36	0.02
PG34:0	3.30	0.00			
PG34:1	3.25	0.00			
PG34:2	2.04	0.01			
PG34:4	2.76	0.00			
PG34:5	3.26	0.00			
PG36:2	2.06	0.05			
PG36:6	2.05	0.04			
PI 31:1p; PI 16:0p/16:0	2.06	0.01			
PI 36:3; PI 16:0/20:3 or 18:0/18:3 or 18:1/18:2	10.07	0.00			
PS30:0	5.24	0.00			
PS30:1	11.23	0.00			
PS31:0	4.17	0.01			
PS31:1	3.87	0.01			
PS32:1	5.30	0.05			
PS33:2	2.03	0.03			
PS34:2	2.03	0.00			
PS34:3	2.97	0.00			
PS34:4	2.90	0.00			
PS34:5	2.79	0.00			
PS35:3	2.19	0.01			
PS36:5	2.69	0.02			
PS36:6	2.49	0.01			
PS37:6	3.11	0.00			

For Review Only
